# Multi-laboratory comparisons of manual patch clamp hERG data generated using standardized protocols and following ICH S7B Q&A 2.1 best practices

**DOI:** 10.1038/s41598-025-15761-8

**Published:** 2025-08-16

**Authors:** Claudia Alvarez Baron, Jun Zhao, Huimei Yu, Ming Ren, Nicolas Thiebaud, Donglin Guo, Giri Vegesna, Cheng-Hui Hsiao, Ryan DePalma, Sabyasachy Mistry, Isra Tariq, Md Shadiqur Rashid Roni, Omnia A. Ismaiel, Murali K. Matta, Vikram Patel, Manni Mashaee, Jose Vicente, Lars Johannesen, Jiansong Sheng, Simon Hebeisen, James Kramer, Andrew Bruening-Wright, Koji Nakano, Hiroshi Matsukawa, Jennifer Beck Pierson, Wendy W. Wu

**Affiliations:** 1 Division of Applied Regulatory Science, Office of Clinical Pharmacology, Center for Drug Evaluation and Research, US Food and Drug Administration, Silver Spring, MD USA; 2 Division of Cardiology and Nephrology, Office of Cardiology, Hematology, Endocrinology and Nephrology, Office of New Drugs, Center for Drug Evaluation and Research, US Food and Drug Administration, Silver Spring, MD USA; 3 CiPA Lab, Gaithersburg, MD USA; 4 B’SYS GmbH, Witterswil, Switzerland; 5 Charles River Laboratories, Cleveland, OH USA; 6 Drug Safety Testing Center Co., Ltd., Higashimatsuyama Laboratories, Saitama, Japan; 7 Health and Environmental Science Institute, Washington, DC USA; 8 Present Address: Division of Pharmacology/Toxicology Review, Office of Safety and Clinical Evaluation, Office of Generic Drugs, Center for Drug Evaluation and Research, US Food and Drug Administration, Silver Spring, MD USA; 9 Present Address: Vertex Pharmaceuticals (Europe) Ltd, Abingdon, Oxfordshire UK; 10 Present Address: Division of Nonclinical Science, Office of Science, Center for Tobacco Products, US Food and Drug Administration, Silver Spring, MD USA; 11 Present Address: Department of Pharmacokinetics, Dynamics, Metabolism, and Bioanalysis, Merck & Co., Inc., West Point, PA USA; 12 Present Address: Translational Cures, Washington, DC USA

**Keywords:** Cardiac safety, Delayed ventricular repolarization, Experimental uncertainty, Concentration-inhibition, Assay reproducibility, Safety margin, Drug safety, Electrophysiology

## Abstract

Acute block of hERG channels is the most common mechanism underlying drug-induced QT_C_ prolongation and potentially fatal *Torsade de Pointes* arrhythmia. Updates to ICH E14 Q&As now allow for using negative nonclinical data, including hERG, to support QT_C_ risk assessment in late-stage clinical development. To interpret the hERG results, understanding hERG assay reproducibility or hERG data variability is pivotal. Protocol and best practice recommendations have been provided with the goal of minimizing lab-to-lab data differences, but the impact remains unclear. To fill this knowledge gap, hERG data from a HESI-coordinated multi-laboratory study were leveraged. Using standardized protocol and following best practices for patch clamp studies, five laboratories tested 28 drugs using the manual patch clamp technique. Systematic differences in block potencies were observed for data generated by one laboratory for the first 21 drugs, and these differences disappeared for the last seven drugs. Exposure, pharmacological sensitivity of the cell lines, and cell/data qualities were ruled out as the factors underlying systematic differences. All laboratories retested two drugs and obtained results within 1.6X of the initial testings, except for another laboratory that obtained data for one drug that differed from its initial testing by 7.6X. Descriptive statistics and meta-analysis were applied to the dataset to estimate what the distribution in hERG block potencies would be if a laboratory were to test the same drug repeatedly. This measure, or hERG data variability, was ~ 5X. Based on these results, hERG block potency values within 5X of each other should not be considered different, since these values are within the natural data distribution of the hERG assay; laboratory-specific safety margin threshold may be required to account for systematic data differences.

**Supplementary Information:**

The online version contains supplementary material available at 10.1038/s41598-025-15761-8.

## Introduction

Block of hERG channels can prolong the cardiac action potential, leading to delayed repolarization that manifests as QT_C_ prolongation and can result in the rare but potentially fatal ventricular tachyarrhythmia *Torsade de Pointes* (TdP). Therefore, the International Council for Harmonization (ICH) S7B guideline recommends testing for hERG block^1^and ICH E14 describes the conduct of dedicated clinical QT studies to assess the potential of QT_C_ prolongation and inform the intensity of ECG monitoring in late-stage clinical development and labeling^2^.

Since implementation of both ICH guidelines, accumulating evidence continues to show that hERG block by small molecules is the most common mechanism of drug-induced QT_C_ prolongation and TdP. This knowledge led to updates of two ICH E14 Questions and Answers (Q&As) that allow for using nonclinical data, including hERG, generated following best practices described in ICH S7B Q&As to facilitate QT_C_ and proarrhythmia risk assessment in late-stage clinical development^3^. Prior to these updates, such risk assessment mainly relied on clinical data.

A negative hERG assay, suggesting that the drug has a low likelihood of hERG block-mediated QT_C_ prolongation and TdP, is defined as having a safety margin (i.e., concentration that inhibits 50% of hERG current (IC_50_) divided by relevant clinical exposure level) larger than the margin threshold derived from a series of reference drugs with known TdP risk^3^ (see ICH S7B Q&A 1.1). Defining a hERG safety margin threshold using literature data pooled across studies is challenging. Many studies were conducted with protocol variations known to impact hERG block potency, including the use of different recording temperatures^4–7^stimulation frequencies^7–10^and voltage waveforms^4,6,8,9^. Drug loss during patch clamp experiments can happen^11–13, ^yet most studies did not verify drug exposure to the cells (i.e., drug concentrations). On the clinical side, high quality ECG data are needed from many reference drugs to understand the distribution of margin threshold for hERG blockers, and this information is also not available in the literature. Even if a margin threshold could be defined using literature data, several questions remain. Can this margin threshold be used to interpret hERG results from all laboratories? The study by Hanson et al. that showed systematically different hERG block potencies from two laboratories suggests not^14^. How about variability in hERG block potency? Are numerical differences in hERG block potencies (hence safety margins) for two drugs reflective of natural distribution (i.e., data distribution that results from testing the same drug over and over again) or real block potency differences between the two drugs? Answers are needed here to lend credibility to the hERG data to support integrated nonclinical-clinical risk assessment as presented in the updated ICH E14 Q&As.

Several efforts have been made to reduce cardiac ion channel data variability that arise from the use of different protocols and generate a new dataset to support further use of nonclinical data in decision-making. ICH S7B Q&A 2.1 was developed to recommend best practices for patch clamp assays, including hERG. These best practices include using more physiologically relevant protocols and concentration verification in an attempt to improve nonclinical-clinical translation. The FDA also released a hERG assay protocol to support assessment of hERG block-mediated QT_C_ prolongation risk (https://www.fda.gov/media/151418/download). The impacts of these efforts remain to be evaluated.

In 2019, a global, multi-laboratory research project, coordinated by the International Life Sciences Institute (ILSI)—Health and Environmental Sciences Institute (HESI), was launched to quantify block of cardiac Na^+^, Ca^2+^, and hERG channels by 28 drugs following best practices and using the FDA protocol, to support continued development of in silico risk prediction models as a part of the Comprehensive in vitro Proarrhythmia Assay (CiPA) initiative^15^. The hERG data generated by the participating five laboratories operating manual patch clamp systems are presented here to demonstrate real world data variabilities both across and within laboratories. Importantly, how these results could be considered when determining whether a molecule carries hERG block-mediated QT_C_ prolongation risk is discussed. Data are fully available at https://osf.io/a6k5t/ to facilitate future research efforts directed at advancing cardiac safety, including derivation of hERG safety margin.

## Materials and methods

### Participating laboratories, experimental protocols, and study design

In alphabetical order, the participating laboratories were: B’SYS GmbH (Switzerland), CiPA Lab, LLC (USA), CRL (Charles River Laboratories, USA), DSTC (Drug Safety Testing Center Co., LTD., Japan), and FDA (Food and Drug Administration, USA). Information and data from individual laboratories are presented in an anonymized fashion, except for the drug source (Supplemental Table 1).

All laboratories used the same voltage waveform and internal and external solutions to record the hERG current at near physiological temperature. The original protocol document shared with participating laboratories may be found at https://osf.io/a6k5t/. This protocol document was subsequently updated to incorporate best practices in the ICH S7B Q&A 2.1 (https://www.fda.gov/media/151418/download). Note that the update did not impact the ion channel protocols as these were already consistent with best practices. The cell lines, drug sources, drug stock (i.e., stock solution) preparation and drug solution handling procedures, including methods of drug delivery to the recorded cells, were not standardized. Test concentrations for each drug were not defined. Instead, each laboratory was asked to test at least four concentrations that yielded good coverage of the concentration-inhibition relationship unless solubility limit was reached. As this study progressed, a separate research project by the FDA found that some drugs in this study can show substantial loss in the FDA’s manual patch clamp systems^16^. Therefore, a bioanalysis arm was added to estimate loss of individual drugs in each laboratory’s custom-built perfusion systems.

The study initially involved measuring 14 drugs’ hERG block potencies for across-laboratory comparisons, followed by repeat testings of two drugs to assess within-laboratory variability (Phase 1). Subsequently, 14 more drugs were added to the study (Phase 2). Lab 3 did not participate in Phase 2. Phase 1 and 2 drugs were tested in a blind fashion by Labs 1 and 4, and open-label for other laboratories, except for repeat testing for which all laboratories used blinded drug stock (see “Repeat testing of drugs during Phase 1” below). hERG block potency data for four drugs (cisapride, dofetilide, terfenadine, and sotalol) by the FDA were previously published^16^.

### Cell lines

Each laboratory used its own cell line that stably expresses the hERG1a subunit. Labs 2 to 5 used human embryonic kidney 293 cells (HEK 293). Lab 1 used Chinese hamster ovary cells (CHO) (Supplemental Table 2). Each laboratory followed its own cell culture procedures.

### Electrophysiology and drug application

Experiments were conducted using the manual whole-cell patch clamp method. For Labs 1 and 4, the recording temperature was 36 ± 1 °C; for Lab 2, 35 to 38 °C; and for Labs 3 and 5, 37 ± 2 °C. Temperature was monitored throughout the recordings with a thermistor placed in the recording chamber.

The recording chamber with cells was continuously perfused with external solution for baseline recording using a gravity-fed system with a flow rate of 1 to 1.5 mL/min for Lab 1, 2 mL/min for Lab 2, 0.5 to 2 mL/min for Lab 3, and 1.5 to 3 mL/min for Lab 5, or a peristatic pump-driven system at flow rate of 5 mL/min for Lab 4. Individual drugs were then applied as the recordings continued using the same perfusion method used for baseline recording for Labs 1, 3, 4, and 5. For Lab 2, perfusion of the external solution was stopped just prior to drug application, and drug solution, preheated in a water bath to 37 °C, was delivered to the recording chamber via pipetting. For Lab 2, depending on the kinetics of block for individual drugs, approximately 2 to 5 mL of drug solution were added to a recording chamber that could hold ~ 500 µL of solution, and excess fluid was removed via a suction tube placed in the recording chamber.

The external solution contained (in mM): 130 NaCl, 5 KCl, 1 MgCl_2_·6H_2_O, 1 CaCl_2_·2H_2_O, 10 HEPES, 12.5 dextrose; pH adjusted to 7.4 with 5 M NaOH; ~280 mOsm/L. The internal solution contained (in mM): 120 K-gluconate, 20 KCl, 10 HEPES, 5 EGTA, 1.5 MgATP; pH adjusted to 7.3 with 1 M KOH; ~280 mOsm/L. Except for some recordings by Lab 1, the voltage command values for all laboratories were corrected for the 15 mV liquid junction potential that resulted from using the above external and internal solutions at 37 °C, estimated using pClamp 10 software (Molecular Devices, CA, USA). Outward current was evoked using a “step-ramp” voltage waveform aimed at mimicking a ventricular action potential: from −80 mV, the membrane was depolarized to +40 mV for 500 ms, followed by a voltage ramp down to −80 mV in 100 ms (−1.2 V/s) (Supplemental Fig. 1A). The stimulation frequency was 0.2 Hz. A hyperpolarizing voltage step from −80 to −90 mV for 100 ms preceded the depolarizing voltage step, and the resulting current was used to calculate the resting input resistance (R_input_) throughout the experiment. The amplifiers and digitizers used by the laboratories are as follows: Lab 1, EPC 10 USB (HEKA, an affiliate of Harvard Bioscience Inc., MA, USA); Lab 2, MultiClamp 700A (Molecular Devices, CA, USA) and National Instruments BNC-2110 and PCI-6221 DAQ (National Instrument, TX, USA); Lab 3, Axopatch 200B, MultiClamp 700B, Digidata 1320A, and Digidata 1332A (Molecular Devices, CA, USA); Lab 4, Axopatch 200B and Digidata 1550A (Molecular Devices, CA, USA); and Lab 5, MultiClamp 700B and Digidata 1550A (Molecular Devices, CA, USA). Series resistance was electronically compensated at the following percent: Lab 1, 50 to 70%; Lab 2, 60%; Lab 3, 0%; Lab 4, 80%; and Lab 5, 80%.

At the end of each recording, the hERG channel blocker E-4031 was applied to each cell to eliminate the hERG current completely (Supplemental Figs. 1B, 1D, 2A, 2C, and 2E). Labs 1, 2, 4, and 5 used 1 µM E-4031; Lab 3 used 0.5 µM.

### Drugs

Phase 1 drugs are ranolazine, terfenadine, dofetilide, chlorpromazine, verapamil, metoprolol, tamoxifen, ondansetron, cisapride, clozapine, pimozide, sotalol, azimilide, and disopyramide. Phase 2 drugs are astemizole, bepridil, vandetanib, ibutilide, pitolisant, domperidone, droperidol, quinine, hydrodolasetron, clarithromycin, moxifloxacin, diltiazem, risperidone, and mexiletine. Drugs and E-4031 were obtained from Sigma-Aldrich (MO, USA), Selleckchem (Germany), Tocris Bioscience (MN, USA), AdooQ Bioscience (CA, USA), Toronto Research Chemicals (ON, Canada), or FUJIFILM Wako Pure Chemical (Japan). Supplemental Table 1 shows the source and catalog number of each drug for each laboratory. Note that the sources of metoprolol were Sigma-Aldrich and Tocris Bioscience. The molecular weight provided by Sigma-Aldrich is for metoprolol monomer (342.4 g/mol); by Tocris Bioscience, metoprolol dimer (684.8 g/mol). In this manuscript, metoprolol potency estimations were based on the molecular weight of the monomer.

### Repeat testing of drugs during Phase 1

Dofetilide and ondansetron were chosen for repeat testing because bioanalysis of their stock solutions in DMSO indicate that they would be stable, thereby mitigating the concern of drug degradation during the shipping process. Stock solutions were prepared by the FDA and shipped on dry ice to each laboratory. These stocks were stored at −80 °C until use. Four concentrations of stock solutions were supplied per each drug, for 1:1000 dilution into external solution to prepare drug solutions for testing.

Stocks were tested in a blind fashion by all laboratories including the FDA. Each laboratory analyzed the recorded traces to estimate fractional hERG inhibition per cell for each stock, and the results were provided to the FDA for unblinding and estimating drug potencies. For Labs 1, 2, 3, and 5, repeat testing occurred approximately two years after initial testing; for Lab 4, approximately seven months.

### Sample collection for liquid chromatography-tandem mass spectrometry (LC-MS/MS) analysis

Samples for all Phase 1 drugs from all laboratories were collected in satellite experiments. In satellite experiments, the drug perfusion apparatuses were set up as with real experiments for near physiological temperature recording, but no patch clamp recordings took place. Samples were collected for all four-to-five drug concentrations tested in the patch clamp experiments, before and after delivery to the recording chamber to measure drug concentrations and estimate drug loss in the system. Samples before delivery (*starting*) were taken directly from the container used to prepare the drug solution at room temperature. Samples after delivery (*final*) were taken directly from the recording chamber. For clozapine, tamoxifen, and pimozide, 100 µL samples of each concentration were collected in triplicate and placed into 2 mL low-binding tubes (0030108450, Eppendorf, CT, USA) to minimize nonspecific binding. For other Phase 1 drugs, triplicate 200 µL samples were collected and placed into 600 µL polypropylene tubes (05-408-120, Fisher Scientific, MA, USA). Labs 1, 2, and 5 collected samples using the same apparatus used for patch clamp recordings. Labs 3 and 4 used a replica perfusion apparatus of the same materials and configuration as the recording apparatus. For Labs 1, 3, 4, and 5, samples were collected after 5 min of perfusion with each drug concentration. For Lab 2, 2 mL of drug solution preheated to 37 °C were directly pipetted into the recording chamber filled with external solution (with suction engaged to remove excess fluid), and samples were collected from the recording chamber after 5 min of incubation. All samples were immediately frozen after collection, transferred to a −80 °C freezer for storage, and then shipped frozen on dry ice to the FDA for bioanalysis.

During Phase 2, Labs 1, 4 and 5 continued to collect samples in satellite experiments. Based on the lessons learned from Phase 1 bioanalytic work, triplicate samples of 200 µL each were collected and placed in tubes containing 400 µL of 5% bovine serum albumin (BSA) solution in water (w/v) to protect samples from nonspecific binding to the collection tubes and degradation during processing. Lab 2 collected samples during the real electrophysiology experiments performed to measure drug potencies. In Lab 2’s experiments, a portion of the drug solution to be tested was used to prepare the drug + 1 µM E-4031 solution used to eliminate the hERG current at the end of the experiments. Final samples obtained from the recording chamber at the end of each electrophysiology recording hence contained both the drug and E-4031. As for other Labs, starting samples were taken directly from the container used to prepare the drug solution at room temperature to enable calculations of percent drug loss. Like Labs 1, 4, and 5, Lab 2 also collected triplicate samples in 5% BSA (w/v) for Phase 2 drugs. Lab 2 collected 100 µL samples into tubes containing 200 µL of BSA solution.

### LC-MS/MS bioanalysis

Methods were developed and validated according to the FDA bioanalytical method validation guidance^17^ for target analytes in hERG external solution for Phase 1 and hERG external solution with 5% BSA solution (1:2 v/v) for Phase 2. Mexiletine was the exception, which had the hERG external solution and 5% BSA ratio of 1:4 (v/v). Methods for cisapride, dofetilide, sotalol, and terfenadine were previously published^18^. Linearity ranges for each drug are provided in Supplemental Table 10. Dilution integrity for samples above the calibration curve was established based on the expected highest concentration for each analyte. Precision and accuracy were established over at least three validation runs. Bench top stability in the hERG external solution (Phase 1) or hERG external solution with 5% BSA (Phase 2) at room temperature (with and/or without organic solvent treatment) were evaluated for analytes except verapamil, which was processed on ice and in the dark to minimize light-dependent degradation. Stability of processed samples at 2 to 8 °C, freeze/thaw stability, and long-term stability (that covered storage duration of samples) at −80˚C were also conducted. Sample preparation was optimized for each analyte, and dilution with organic solvents (acetonitrile or methanol) was applied for all analytes. To minimize instability, cisapride, terfenadine, and dofetilide samples were treated with 5% BSA (w/v); chlorpromazine samples were thawed in the presence of twice the amount of organic solvent (acetonitrile). Samples for other Phase 1 analytes with significant nonspecific binding (i.e., tamoxifen, clozapine, and pimozide) were collected in low-binding tubes and treated with organic solvent(s) in the collection tube to minimize drug loss. Isotopically labeled internal standards were used for all analytes, except for the following for which the internal standard used is indicated in parenthesis: disopyramide (clozapine-d_4_), bepridil (clarithromycin-d_3_), domperidone (astemizole-d_3_), droperidol (diltiazem-d_3_), hydrodolasetron (diltiazem-d_3_), ibutilide (quinine-d_3_), vandetanib (quinine-d_3_), and pitolisant (moxifloxacin-^13^C-d_3_).

Agilent Ultra-Performance Liquid Chromatography (UPLC) and a SCIEX 6500 + triple quadrupole/Q-trap or SCIEX 4500 mass spectrometer with positive mode electrospray ionization were utilized for detection of all analytes except for ranolazine and chlorpromazine. For other Phase 1 analytes, chromatographic separation was achieved on a Phenomenex Kinetex XB-C18 column (2.1 × 50 mm; 1.7 μm) at room temperature. Gradient elution using 10 mM ammonium formate in 0.1% formic acid in water (v/v) (solvent A), and acetonitrile with or without 0.1% formic acid (v/v) (solvent B) was applied. Waters Xevo Acquity UPLC in positive ionization mode was utilized for ranolazine and chlorpromazine. Chromatographic separation was achieved on a Waters Acquity HSS T3 column (2.1 × 50 mm; 1.8 μm) using 0.1% formic acid in water (v/v) (solvent A), and acetonitrile (solvent B). For Phase 2 analytes, chromatographic separation was achieved on a Waters Acquity BEH C18 column (2.1 × 100 mm; 1.7 μm) for all drugs except domperidone, for which Waters Acquity HSS T3 column was used. Gradient elution using 0.1% formic acid in water (v/v) (solvent A), and 0.1% formic acid in acetonitrile (v/v) (solvent B) was applied for all analytes except bepridil. For bepridil, the mobile phases used were as follows: aqueous mobile phase was 10 mM ammonium formate in water (solvent A), and the organic mobile phase was 0.1% formic acid in acetonitrile (v/v) (solvent B). Chromatographic conditions and mass spectrometry parameters were optimized individually for each analyte. Selective LC-MS/MS methods were developed, with no interference (< 20% of lower limit of quantification or LLOQ) for individual target analytes. Absence of interference by E-4031 on the quantification of Phase 2 drugs was confirmed during method validation.

### Estimation of drug potencies

Drug inhibition of hERG current was measured at the outward ramp current elicited by the repolarizing voltage ramp. Current traces or peak amplitude of the ramp current measured in E-4031 were subtracted from the recorded traces or absolute peak amplitude of the ramp current (different approaches reflect laboratory-specific practices for current subtraction) to isolate the E-4031-sensitive, presumably hERG current that was then used to calculate fractional inhibition by each drug concentration. Supplemental Figs. 1C, 1E, 2B, 2D, and 2F show time course plots of the E-4031-subtracted hERG current amplitude (ramp current, *top panel*), current required to clamp the cell at −80 mV (I_−80 mV_; *middle panel*), and R_input_ (*bottom panel*). In a limited number of cells, E-4031 was not added, and the absolute ramp current amplitude was used to calculate fractional inhibition. Each laboratory analyzed its own recorded traces to estimate fractional inhibition per drug concentration per cell by calculating $\:(1-\:\frac{hERG\:current\:in\:drug}{hERG\:current\:in\:baseline}$). When estimating hERG block potencies, nominal drug concentrations were corrected by the FDA for the losses observed in satellite experiments for all laboratories during Phase 1. Firstly, fractional drug loss in satellite experiment was calculated from the average of the triplicate samples for each concentration using: $\:1-\:\frac{{\left[\text{D}\text{r}\text{u}\text{g}\right]}_{final}}{{\left[\text{D}\text{r}\text{u}\text{g}\right]}_{starting}}$ (see *“Sample collection for liquid chromatography-tandem mass spectrometry (LC-MS/MS) analysis”* for definitions of *“starting”* and *“final”* samples*)*. Then, nominal concentrations were multiplied by (1 − fractional drug loss) to estimate the remaining drug concentrations. For most drugs, fractional drug loss was determined by averaging losses from samples across all concentrations for that drug. For the eight drugs from specific laboratories that showed a trend for concentration-dependent drug loss in satellite experiments (Supplemental Table 3), each nominal concentration for that drug was corrected using the fractional drug loss for that specific concentration (Supplemental Fig. 4A and 4B). Exception to this approach of concentration correction was the blind repeat testing of dofetilide by Labs 2 and 4. Data from these two laboratories showed concentration-dependent loss for dofetilide. Given that blind repeat testing used different nominal concentrations prepared by the FDA, correcting fractional loss by concentration was not possible. For the blind repeat testing data, drug potency values for these laboratories were corrected using averaged loss across all concentrations. For Lab 2 during Phase 2, the analytical concentrations measured for individual cells were used as corrected concentrations to estimate drug potencies.

Corrected concentrations were used to generate concentration-inhibition plots using the fractional hERG inhibition values for each cell reported by each laboratory. Concentration-inhibition plots were then fitted with the Hill equation with variable slope to estimate hERG block parameters and 95% confidence interval (CI): Fractional inhibition = $\frac{1}{1+{(\frac{{IC}_{50}}{[drug]})}^{nH}}.$ Here IC_50_ is the concentration that inhibited 50% of the current, [drug] is the drug concentration, and n_H_ is the Hill coefficient. The pIC_50_ values with 95% CI were estimated by converting the corrected concentrations expressed in molar to the common logarithm, and then fitting the concentration-inhibition plots with the Hill equation. Supplemental Fig. 4C illustrates how these values were estimated. Hill fits and pIC_50_s conversions were done using Igor Pro 8.0 (Wavemetrics, USA).

### Estimating variability in hERG block potency

Two methods—descriptive statistics and meta-analysis—were used to estimate variability in hERG block potency. Both methods attempt to attribute pIC_50_s derived from individual experiments into two sources: (1) *real effect* due to drug- and laboratory-specific impacts; and (2) *residual variability* (or unexplained variability), the distribution of which, when aggregated across experiments, defines variability in hERG block potency. The descriptive statistics approach calculated the center of distribution for each drug’s pIC_50_s as the average of all laboratories’ pIC_50_s for that drug without accounting for within-experiment variability that results from fitting fractional inhibition values from different cells in the concentrate-inhibition plots. Then, the “distances” of each laboratory’s pIC_50_s to the centers of distribution for all drugs were calculated, effectively removing drug-specific impacts. Averaging these distances yielded a fixed, laboratory-specific impact, which when removed, revealed the residual variability for individual experiments. Details of this approach are explained in the “Results” section along with a “step-by-step” figure.

Variability in hERG block potency was also assessed in R 4.2.2 (R Foundation for Statistical Computing, Vienna, Austria) using a mixed-effects meta-analysis framework implemented with the “location-scale” model in the metafor package version 4.0.0^19,20^. Pooling data across laboratories and across drugs, a standard mixed-effects model was used to estimate variability in hERG block potency in a laboratory-agnostic fashion, while the location-scale model was used to assess laboratory-specific variability in hERG block potency. Both models assumed that pIC_50_ values are the sum of drug- and laboratory-specific impacts, residual variability, and within-experiment variability (also thought of as measurement errors). The general formula is as follows:

$$\:pI{C}_{{50}_{i,j,k}}=dru{g}_{i}+laborator{y}_{j}+{u}_{i,j,k}+{\varepsilon}_{i,j,k}$$

Here $\:{pIC}_{{50}_{i,j,k}}$ represents the observed pIC_50_ value for drug *i*, tested in laboratory *j*, and in experiment *k*. Drug_*i*_ and laboratory_*j*_ are fixed effects. $\:{u}_{i,j,k}$ is a random effect capturing residual heterogeneity or between-study variability and is represented by a normal distribution centered at 0 with a residual variance $\:({\uptau}$^2^). Thus, $\:{u}_{i,j,k}\sim\:N\left(0,{\tau}^{2}\right)$. $\:{\varepsilon }_{i,j,k}\:$is the within-experiment measurement error assumed to follow a normal distribution and is represented by $\:{\varepsilon }_{i,j,k}\sim\:N\left(0,{\sigma}^{2}\right)$, where $\:\sigma\:$ is the standard deviation (SD) of $\:{pIC}_{{50}_{i,j,k}}$. A key difference between the mixed-effects model and the location-scale model is the handling of residual variance $\:{\uptau}$^2^. The mixed-effects model assumes that the residual variability is the same across all drugs and all laboratories. The location-scale model makes the same assumption for drugs but allows the residual variability to differ by laboratory: $\:{u}_{i,j,k}\sim\:N\left(0,{\tau}_{j}^{2}\right)$. Thus, each laboratory *j* has a unique residual variance $\:{\tau}_{j}^{2}$ to reflect laboratory-specific experimental procedures and conducts. A table containing the input data for the meta-analysis may be found at https://osf.io/a6k5t/.

Variability in hERG block potency is expressed as the ratio of the 97.5th and 2.5th percentiles of the residual variability and is calculated as follows: the 95% CI is calculated by multiplying the SD from the mixed-effects and location-scale models ($\:\tau\:$ or $\:{\tau}_{j}$) by the 97.5th percentile of a standard normal distribution ($\:qnorm\left(0.975\right)=\:\sim\:1.96$), then multiplying that result by two to account for the full spread (± 1.96 SD), thus converting the SD into a range that spans from the 2.5th to the 97.5th percentile. By exponentiating with base 10, variability is converted from a log-scale measure (pIC_50_) to a ratio of IC_50_. The final formula is: $\:{10}^{\left(2\:\times\:\text{qnorm}\left(0.975\right)\:\times\:\tau\right)}$. Supplemental Fig. 3 shows simulated data that illustrates this process. Panels A to C show three simulated concentration-inhibition curves for a series of hERG experiments, and panels D and E show the corresponding distributions of pIC_50_s and IC_50_s, respectively, which includes the 2.5th and 97.5th percentiles. In the simulated data, the SD of pIC_50_s is 0.20, which translates to a ratio of 6.09.

### Fully automated electrophysiology data analysis and reporting

The original recorded current traces and experimental information were exported and coded following the Tabulated Experimental Data (TED) format, an open data format that includes summary spreadsheets for the experimental conditions and comma-separated value (CSV) files storing the recorded waveforms. TED data sets were then automatically analyzed using custom software in Python version 3.10.4 (Python Software Foundation). Analysis output included figures with original and E-4031 subtracted current traces (Supplemental Figs. 1B, 1D, 2A, 2C, and 2E), and time course plots of cell parameters (ramp current, I_−80 mV_, and R_input_; Supplemental Figs. 1C, 1E, 2B, 2D, and 2F). To facilitate data review, results were collated in an html report for each laboratory and drug in R version 4.2.2. TED datasets, format metadata specifications, analysis (Python), and reporting (R) scripts are available at https://osf.io/a6k5t/. A Python package to read and write TED datasets is also available as open source at https://osf.io/a6k5t/.

## Results

### hERG block potency data for the initial 14 drugs studied

This study focuses on understanding hERG data variability, which is defined by the distribution of hERG block potencies. In main figures, data related to hERG block potencies are illustrated using pIC_50_ values (i.e., -log_10_(IC_50_ in molar)) as these are normally distributed hence more intuitive to relate. In contrast, the more commonly used IC_50_ values are log-normally distributed (Supplemental Fig. 3). When summarizing relationships of hERG block potencies in the text, the differences are presented as ratios of IC_50_s. hERG block estimations also took into account drug loss due to nonspecific binding (see “Methods”). Supplemental Table 4 provides the pIC_50_ values and 95% CI for all drugs by all laboratories. For completeness, Supplemental Tables 5 and 6 provide the values of IC_50_, n_H_, and respective 95% CI estimated using the nominal and corrected drug concentrations, respectively.

**Fig. 1:**
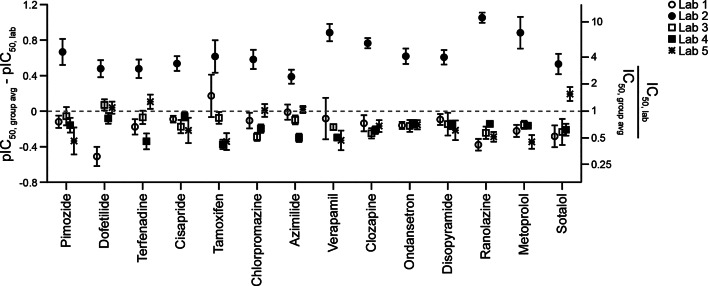
Across-laboratory data comparison for Phase 1 drugs. A plot of the average pIC_50_ value across five laboratories minus individual laboratory’s pIC_50_ for the studied drugs. The left Y-axis is equal to $\:Log\left(\frac{{\text{I}\text{C}}_{50,\:lab}}{{\text{I}\text{C}}_{50,\:group\:avg}}\right)$. Error bars: ± 95% CI of pIC_50_. The right Y-axis shows the corresponding changes in multiples of IC_50_ or $\:\left(\frac{{\text{I}\text{C}}_{50,\:lab}}{{\text{I}\text{C}}_{50,\:group\:avg}}\right)$. Drug potency estimations shown here and rest of the figures accounted for drug loss. The averaged differences in pIC_50_ to the group averages across all Phase 1 drugs are as follows (with 95% CI in parenthesis): Lab 1, −0.16 (−0.07 to −0.24); Lab 2, 0.65 (0.75 to 0.55); Lab 3, −0.15 (−0.10 to −0.20); Lab 4, −0.20 (−0.15 to −0.25), and Lab 5, −0.14 (−0.05 to −0.24). Lab 2 shows systematically higher pIC_50_s for all drugs irrespective of drug potencies.

Figure 1 shows comparisons of each laboratory’s hERG block potencies ($\:{\text{p}\text{I}\text{C}}_{50,\:lab}$) to the group average ($\:{\text{p}\text{I}\text{C}}_{50,\:group\:avg}$) for the initial drugs studied (Phase 1). Differences between two values are often expressed as a ratio, and a similar approach is applied here. The left y-axis in Fig. 1 shows $\:{\text{p}\text{I}\text{C}}_{50,\:group\:avg}-{\text{p}\text{I}\text{C}}_{50,\:lab}$, which is log-transformed $\:(\frac{{IC}_{50,\:lab}}{{IC}_{50,\:group\:avg}}$) (right y-axis). The x-axis lists drugs in descending order of block potency. See Supplemental Fig. 5 for a plot of IC_50_ values for these drugs. Across all Phase 1 drugs, pIC_50_s for Labs 1, 3, 4, and 5 are below the group averages and showed similar differences relative to the group average. The average ratios of laboratory-specific IC_50_ values to the group average are (95% CI in parentheses): Lab 1, 0.7 (0.6 to 0.9); Lab 3, 0.7 (0.6 to 0.8); Lab 4, 0.6 (0.6 to 0.7); and Lab 5, 0.7 (0.6 to 0.9). Lab 2’s hERG block potencies are systematically lower than those of the other laboratories. The averaged ratio of Lab 2 IC_50_s to the group averages for Phase 1 drugs is 4.5 (3.4 to 5.6).

### Additional experiments by Labs 2 and 5 to investigate the sources of systematic data differences

Some laboratory-specific practices and materials were used to generate Phase 1 data. These included methods that could impact drug exposure levels (i.e., differences in drug solution delivery to the recorded cells, drug source and drug stock preparation, and solution heating for near physiological temperature recording), use of cell lines that could have different pharmacological sensitivity, and data quality (i.e., data acceptance) criteria. Thus, additional experiments were conducted by Labs 2 and 5 to evaluate the impact of these factors on drug potencies.

**Fig. 2:**
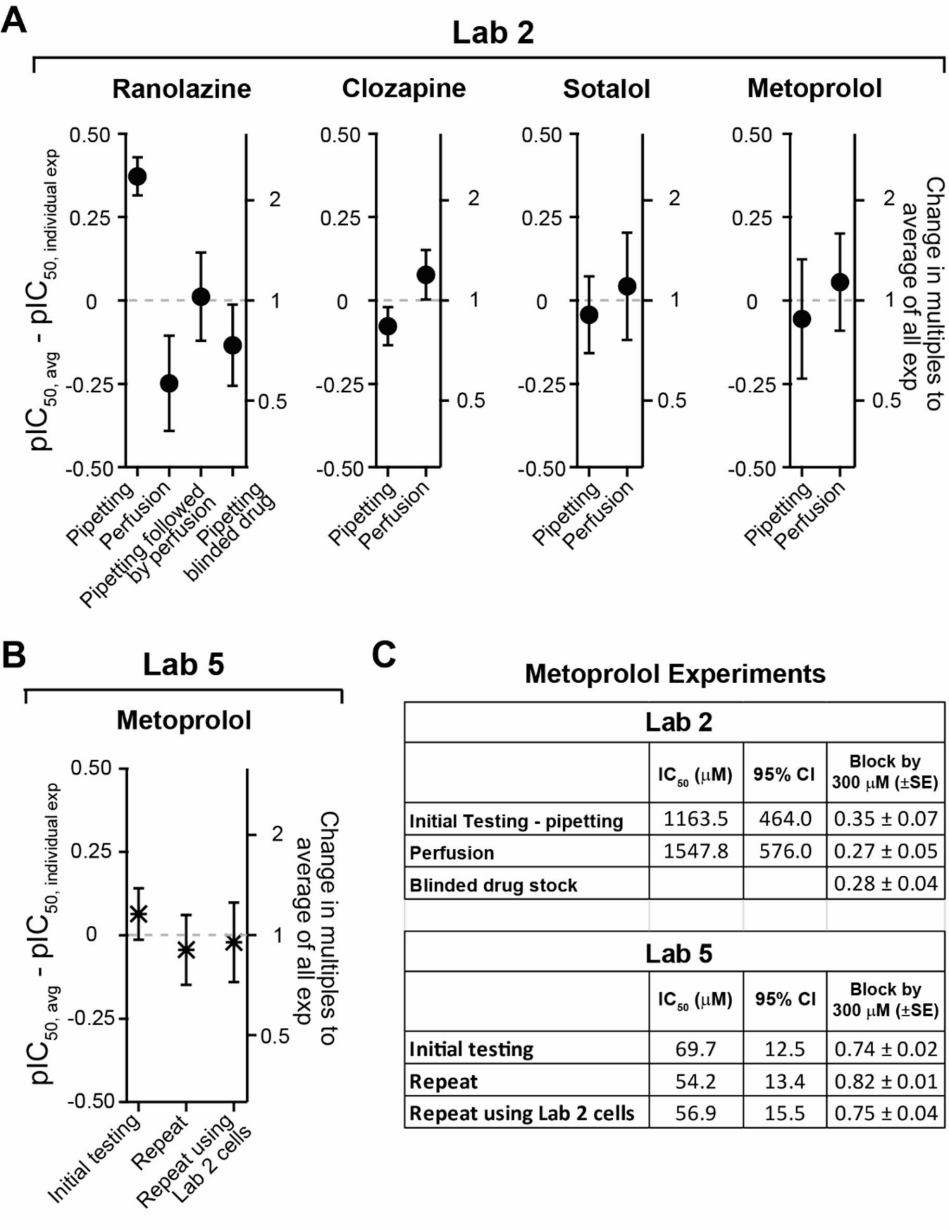
Additional repeat experiments by Labs 2 and 5 to assess impact of drug delivery methods, cell line, and source of drugs/drug stocks on drug potencies. (**A**) Experiments conducted by Lab 2 to assess the impact of delivery method on the potency of ranolazine, clozapine, sotalol, and metoprolol. In the initial testing using the direct pipetting method (Fig. 1), Lab 2’s pIC_50_ differences to the group averages for these drugs were 1.05, 0.76, 0.53 and 0.88, respectively. In additional repeat experiments, drugs were applied using constant perfusion. Plots show the average pIC_50_ values across all experiments for the same drug minus pIC_50_s from individual experiments that tested different drug delivery methods. Error bars: ± 95% CI of pIC_50_ from individual experiments. The pIC_50_ differences to each drug averages are: ranolazine, 0.62; clozapine, 0.15; sotalol, 0.09, and metoprolol, 0.11, (**B**) this panel shows experiments conducted by Lab 5. The plot shows average pIC_50_ values across all metoprolol experiments minus pIC_50_ from individual experiments. (**C**) Summary of metoprolol results by Labs 2 and 5.

Regarding drug delivery methods, Labs 1, 3, and 5 used the gravity-fed, constant perfusion method; Lab 4 used peristatic pump-driven constant perfusion method; and Lab 2 used direct pipetting method to apply preheated drug solutions to the recording chamber. It is often assumed that constant perfusion causes less drug loss than directly applying drug to a recording chamber with static solution because the former pre-saturates nonspecific binding sites in the perfusion apparatus. Although hERG block potencies were estimated after accounting for drug loss in this study, the participating laboratories nonetheless remained concerned that this procedural difference impacted hERG block potencies. To address this concern, Lab 2 used the constant perfusion method to retest ranolazine, clozapine, sotalol, and metoprolol—four drugs for which Lab 2’s IC_50_s were 11.3X, 5.8X, 3.4X, and 7.6X larger than the group averages, respectively (Fig. 1). These results are summarized in Fig. 2A. For ranolazine, an additional experiment tested both drug delivery methods in the same cells (i.e., pipetting first followed by constant perfusion), and the result did not reveal further hERG current inhibition by constant perfusion. The ratio of the largest and smallest IC_50_s for ranolazine was 4. For clozapine, sotalol, and metoprolol, the IC_50_ ratios were 1.4, 1.3, and 1.3, respectively. Lab 2 indicated that these results are within the range of data reproducibility for its patch clamp studies and considered both drug delivery methods to yield similar outcomes.

The second hypothesis tested was that the methods of drug stock preparation and sources of drugs impacted hERG block potencies. Here the FDA provided blinded stocks of ranolazine and metoprolol, and Lab 2 tested these using the direct pipetting method. For ranolazine, the block potency is within the range seen in the other three experiments (Fig. 2A). For metoprolol, an IC_50_ was not estimated because the fractional hERG inhibition was less than 0.5 for the highest concentration provided. Nonetheless, comparison of this fractional inhibition value to those achieved by the same concentration in the initial experiment and the subsequent constant perfusion experiment revealed similar degrees of inhibition (Fig. 2C). These data suggest that the methods of drug stock preparation and sources of drugs are unlikely the cause of systematic data differences by Lab 2.

The third hypothesis tested was that different hERG-expressing cell lines exhibit different pharmacological sensitivity. Here Lab 5 was recruited to perform additional experiments. Firstly, Lab 5 retested metoprolol using its own cell line in an attempt to understand within-laboratory reproducibility (Fig. 2B and C, “Repeat”). Then Lab 5 evaluated Lab 2’s cells (maintained and passaged using Lab 2’s cell culture media; Fig. 2B and C). The results from both experiments were similar to Lab 5’s initial testing, with the difference between pIC_50_s from repeat and initial experiment ($\:{pIC}_{50,\:repeat}-\:{pIC}_{50,\:initial}$) equal to or less than 0.11 (i.e., the ratio of IC_50_ values equal to or less than 1.3). These experiments demonstrate that, at least for metoprolol, Lab 2’s and Lab 5’s hERG-expressing cells did not exhibit different levels of sensitivity.

For near physiological temperature recordings involving the direct pipetting method, Lab 2 maintained drug solutions in a 37 °C water bath for up to 30 min prior to delivery to the recorded cell. For laboratories that used constant perfusion method, drug solutions were transiently heated for a few to tens of seconds using an in-line heater and in some laboratories in combination with a heated recording platform (Supplemental Table 2). The fourth hypothesis tested was that the duration of drug solution heating impacted drug stability hence available drug molecules to block hERG channels. Here the FDA prepared solutions of metoprolol (100 µM), verapamil (1 µM), and clozapine (3 µM), stored samples at 37 °C in a water bath, and collected samples at 0, 15, 30, 45, and 60 min. Samples were placed on ice, and relative concentrations measured using LC-MS/MS. No time-dependent drug loss was found (Supplemental Table 7). These results suggest that the duration of drug solution heating, at least for the drugs tested, was unlikely to contribute to the systematically data differences by Lab 2.

### Repeating dofetilide and ondansetron experiments by all laboratories in a blind fashion

**Fig. 3:**
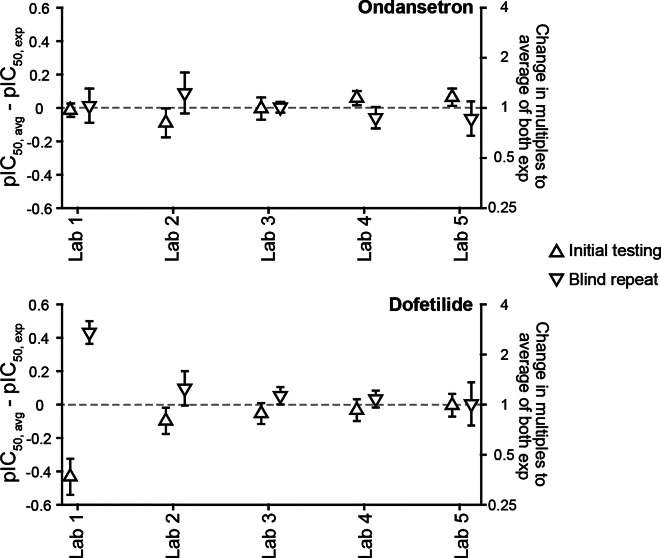
Repeat testings of ondansetron and dofetilide. Plots of averaged pIC_50_s from initial and repeat testings minus pIC_50_ from individual experiments for ondansetron (upper panel) and dofetilide (lower panel). Error bars: ± 95% CI of pIC_50_. The right Y-axes show the corresponding changes in multiples relative to the average of both experiments, or ($\:\frac{{IC}_{50,\_exp}}{{IC}_{50,\_avg}}$).

To understand variability in hERG block potencies obtained by the same laboratory but on different occasions, dofetilide and ondansetron were retested in a blind fashion by all laboratories. Figure 3 shows the differences between the averaged pIC_50_ value from the initial and repeat testing and the pIC_50_ value from individual experiments. For ondansetron, the repeat testing IC_50_ results ranged from 0.6X to 1.6X relative to the initial testing results (Fig. 3, *top panel*). For dofetilide, Labs 2, 3, 4 and 5 generated IC_50_s within 1.5X to those from the initial testings (Fig. 3, *bottom panel*). For Lab 1, the IC_50_ from the repeat testing was 7.6X higher than its initial testing. Dofetilide’s block kinetics on hERG channels is slow, and examination of the time course plots revealed that the recording period for the two lowest concentrations from Lab 1 were insufficient for block development. The two higher concentrations of the repeat testing, however, did show steady state inhibition by dofetilide (see dofetilide blind repeat testing for Lab 1 at https://osf.io/a6k5t/). Thus, dofetilide’s block potency from repeat testing by Lab 1 was also estimated using data from the two highest concentrations only. This resulted in an IC_50_ that is 6.1X larger than the initial testing. Outside this study, Lab 1 repeated the dofetilide experiment again and shared an IC_50_ value of 10.1 nM (n_H_ = 1.6). This value is 2.7X higher than its initial testing and 2.8X lower than the blind repeat testing. Supplemental Table 8 shows the IC_50_ and n_H_ values for these drugs during initial testing and subsequent blind repeat testing. Additional repeat experiments from Labs 2 and 5 for ranolazine, clozapine, sotalol, and metoprolol are also provided.

### Testing of 14 additional drugs

During Phase 1, concentration verification was performed on drug samples collected from satellite and not real patch clamp experiments. Deviations in drug concentrations not attributed to nonspecific binding, including those due to human handling errors and drug instability, would thus not be caught. The potential contribution of this possibility to systematic data differences seen in Phase 1 was evaluated when 14 additional drugs were added to this study (Phase 2). For these drugs, Lab 2 collected drug samples during the real electrophysiology experiments, hence the measured concentrations for individual cells were plotted against fractional current inhibition values to generate the corrected concentration-inhibition graphs to estimate drug potencies. The rest of the laboratories continued to collect satellite samples to assess nonspecific binding-related drug loss to estimate block potencies as described in “Methods” for Phase 1 drugs.

**Fig. 4:**
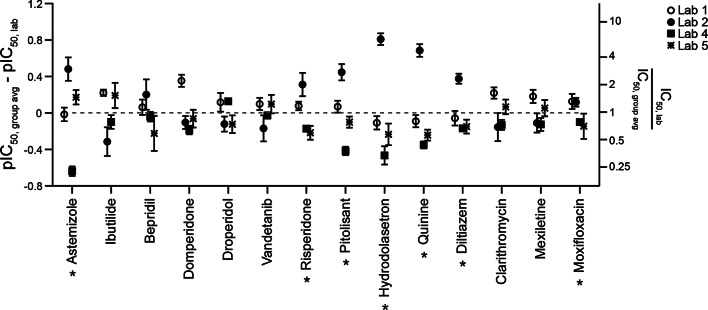
Across-laboratory data comparison for Phase 2 drugs. Same layout as Fig. 1. The averaged pIC_50_ differences to the group averages are as follows: Lab 1, 0.09 (0.02 to 0.16); Lab 4, −0.20 (−0.30 to −0.09), and Lab 5, −0.07 (−0.15 to 0.01). For Lab 2, the averaged pIC_50_ difference to the group averages is 0.46 (0.29 to 0.63) for the first seven drugs tested indicated by an asterisk (*) (astemizole, risperidone, pitolisant, hydrodolasetron, quinine, diltiazem, and moxifloxacin). The systematic differences resolved for the last seven drugs tested by Lab 2 (ibutilide, bepridil, domperidone, droperidol, vandetanib, clarithromycin and mexiletine). The averaged pIC_50_ differences to the group averages for these drugs is −0.11 (-−0.23 to 0.004) for Lab 2.

Figure 4 shows comparisons of each laboratory’s hERG block potencies to the group averages for individual Phase 2 drugs (for IC_50_ values, see Supplemental Fig. 7). The first seven drugs tested by Lab 2 showed lower block potencies relative to group averages, consistent with Phase 1 results. On average, Lab 2’s IC_50_ values are 2.9X (2.0X to 4.3X) larger than the group average for these seven drugs. Unexpectedly, the remaining seven drugs tested by Lab 2 showed no systematic differences. Its averaged IC_50_ values are 0.8X (0.6X to 1.0X) that of the group average for these drugs. The only procedural difference identified by Lab 2 between the two sets of drugs was that the latter seven drugs were tested using cells with lower passage number. A survey amongst the participating laboratories indicated that younger passage number for cell lines has not been observed to impact hERG block potencies. For the other laboratories, the averaged IC_50_ ratios relative to the group averages for Phase 2 drugs are as follows: Lab 1, 1.2 (1.1 to 1.4); Lab 4, 0.6 (0.5 to 0.8); and Lab 5, 0.9 (0.7 to 1.0).

### Drug loss in manual patch clamp experiments

**Fig. 5:**
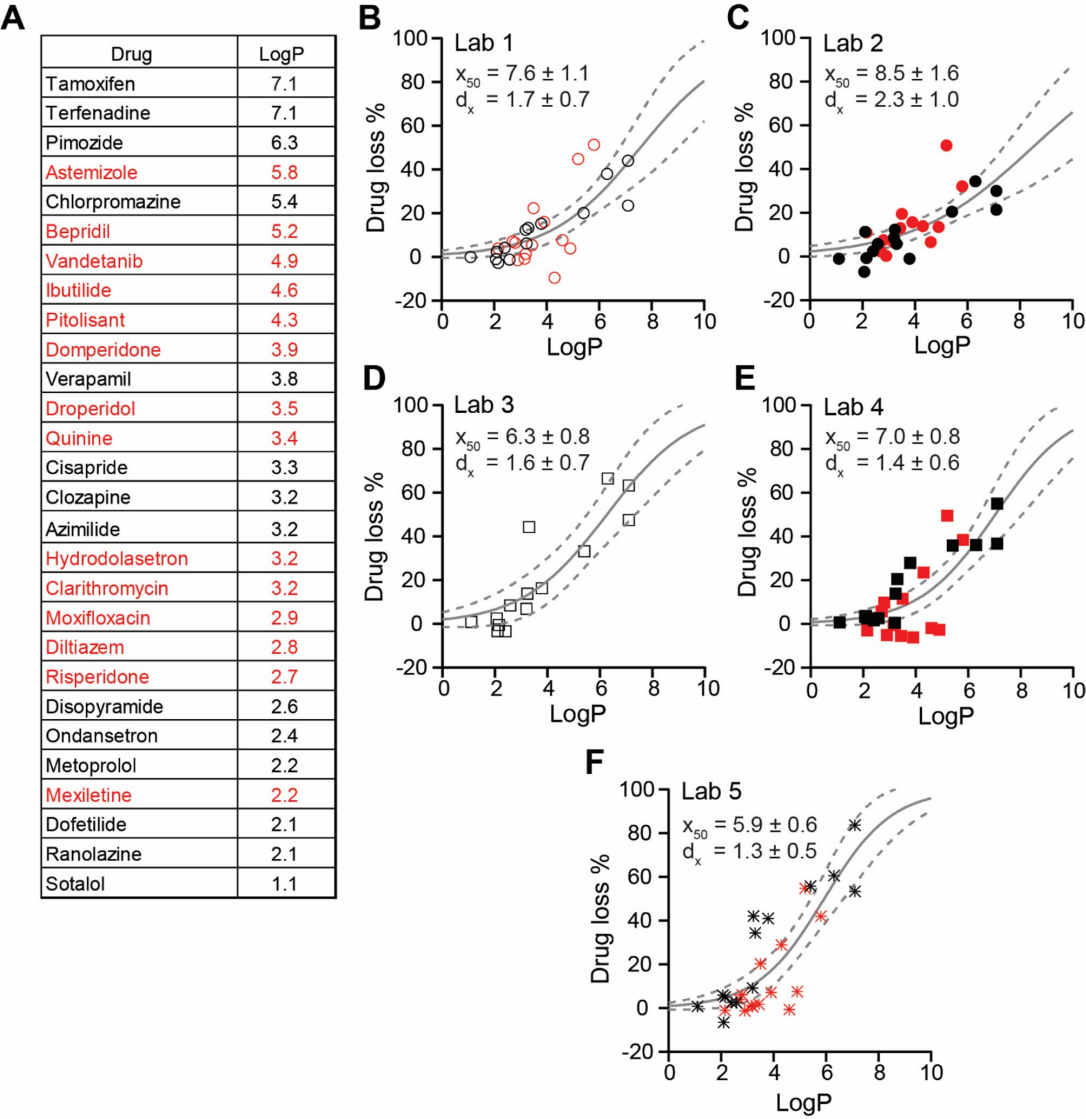
Percent drug loss increases with LogP. Information regarding Phase 1 drugs are shown in black; Phase 2 drugs, red. (**A**) A table of drugs in this study and their respective LogP values. (**B**–**F**) show plots of averaged percent drug loss versus LogP for individual laboratories. A sigmoidal function in the form of $\:y\:=\frac{1}{1+\:{e}^{\frac{{x}_{50}-x}{{d}_{x}}}}$, with minimum and maximum constrained to 0 and 100, respectively, was fitted to the datapoints in the percent drug loss versus LogP plots to yield the LogP at which 50% of the drug is lost (x_50_) and the slope of the relationship (d_x_). The solid gray line shows the fit; the dotted gray lines indicate the upper and lower 95% CI. The coefficient values ± 95% CI are shown in each plot.

Considerable degrees of drug loss were observed in this study, even in satellite samples collected to assess nonspecific binding. Nonspecific binding results from ionic and hydrophobic interactions between the drug molecules and glass and plastic substrates, respectively^21^. The average percent drug loss values (estimated by averaging losses across all concentrations for each drug; Supplemental Tables 9 and 11) were compared with pK_a_ (i.e., the pH at which the ionized and unionized species exist in equal concentrations) and LogP values (i.e., logarithm of the octanol-water partition coefficient that informs the degree of hydrophobicity) of the drugs. No apparent relationship was found with pK_a_ (Supplemental Table 9). However, the extent of drug loss was related to LogP. Figure 5A provides LogP values for the 28 drugs studied, and panels 5B to 5F show the plots of percent drug loss versus LogP for individual laboratories. The percent drug loss increases proportionally with LogP. The plots were fit with a sigmoidal function to illustrate that the extents of drug loss are laboratory-specific. These results suggest that hydrophobic interactions between drug molecules and the manual patch clamp apparatuses used in this study are largely responsible for drug loss due to nonspecific binding.

The average drug losses observed by Lab 2 for Phase 1 and Phase 2 drugs were similar for drugs with similar LogP (Fig. 5C, Supplemental Table 9). Focusing on Phase 2 and considering drugs with similar LogP, the first seven drugs tested by Lab 2 that showed lower block potencies relative to the other three laboratories’ data did not exhibit more drug loss than the latter seven drugs tested that did not show lower block potencies. This comparison continues to support the argument that differences in drug exposure level did not contribute to systematic data differences seen in this study. The concentrations measured in starting and final samples for each drug and laboratory and calculations of percent drug losses can be found in Supplemental Table 11.

### Cell characteristics and data quality

Each laboratory used its hERG-expressing cell line and laboratory-specific data acceptance criteria. The characteristics of the cells recorded by each laboratory, inferred from measurements collected in the vehicle solution, are illustrated with histograms in Supplemental Fig. 6. These measurements include I_−80 mV_, R_input_, hERG current amplitude, and ramp voltage at which hERG current peaked (see “Methods”). Across laboratories, the distributions of these four measurements show considerable overlap—a pattern inconsistent with any measurement being the source of systematic data differences seen for Phase 1 (Fig. 1) and Phase 2 drugs (Fig. 4).

**Fig. 6:**
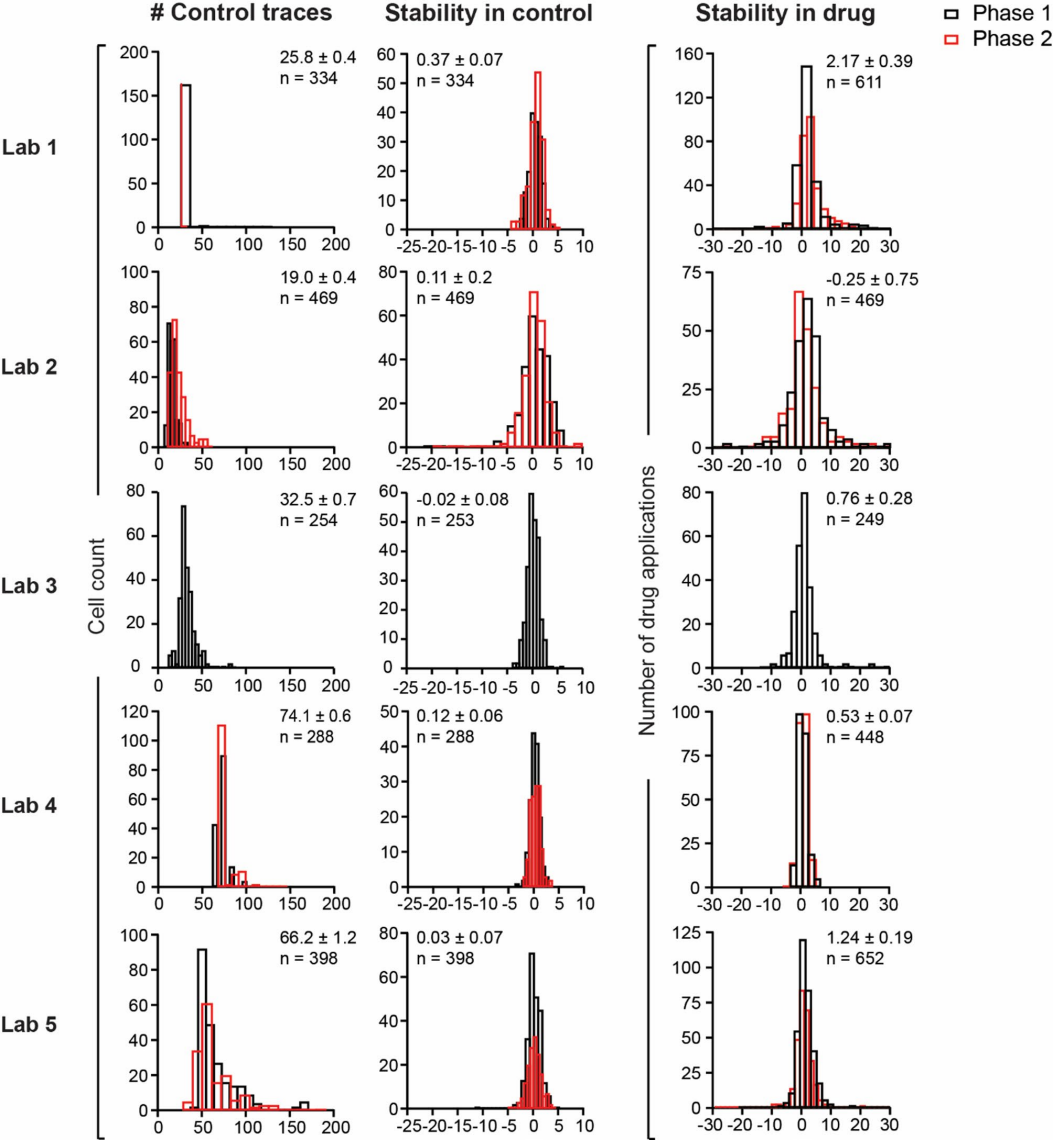
hERG current stability in vehicle control solution and following drug solution application. Histogram plots include all drugs in the study for Phase 1 (black) and Phase 2 (red). The X-axes show number of control traces (left column) or hERG current stability (middle and right columns). Stability in the vehicle (control) and drug solutions were estimated as the percent change between the hERG current amplitudes for the first and fifth trace (20 s apart) of the last five traces recorded in the vehicle or drug solutions, respectively. Labels show mean ± SE of all values. For the histograms illustrating the number of control traces recorded in the control solution and hERG current stability in the control condition, the Y-axes reflect cell count, and the “n” label indicates the total number of cells recorded by each laboratory. For the histograms illustrating the stability of the inhibition achieved following drug application, the Y-axes reflect the number of drug applications, and the “n” label the total number of drug applications. Labs 1, 4, and 5 applied one to two drug concentrations per cell. Labs 2 and 3 applied one concentration per cell.

Data quality is often questioned when unexplained data pattern such as systematic differences emerges. Indeed, strong and continuous current rundown in vehicle and drug solutions can lead to apparent increases in drug block potencies. Conversely, not achieving steady state drug block can lead to apparent decreases in drug block potencies. Figure 6 shows, by laboratory, histograms of the number of control traces recorded in the vehicle solution for each cell, hERG current stability achieved in the vehicle solution (control), and steady state inhibition following drug application. The number of control traces recorded by individual laboratories differed. However, the degrees of hERG current stability, either in the vehicle solution or following drug application, did not reveal distribution shifts that could translate to systematic differences in hERG pharmacology of the magnitude seen in this study. As part of the quality assessment, time-course plots for each parameter of interest for each cell are included in this study (see HTML reports available in https://osf.io/a6k5t/).

### Within-experiment data variability

**Fig. 7:**
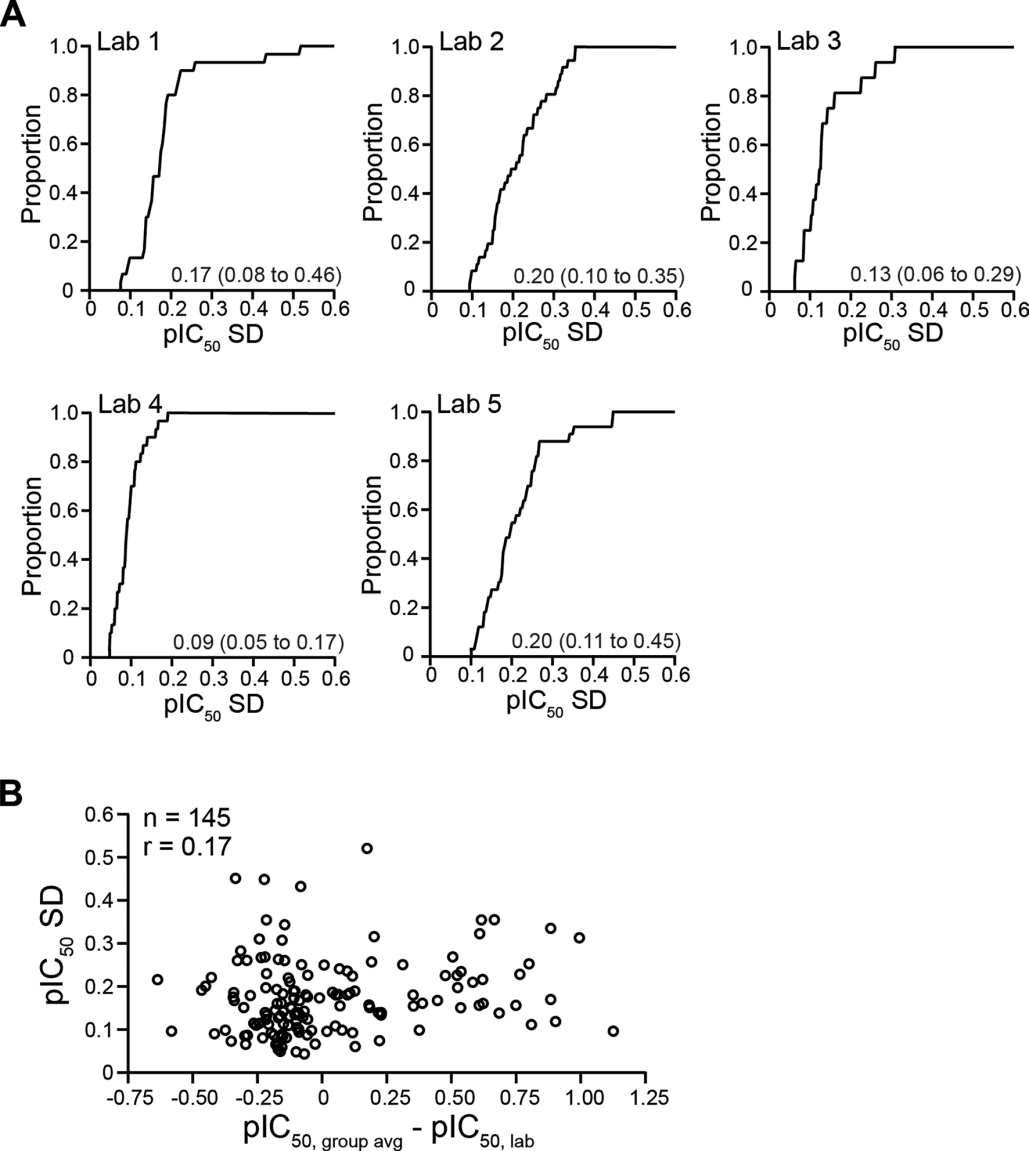
Within-experiment variability. (**A**) Cumulative distribution plots of the SD of pIC_50_s for all experiments, including the initial testings, blind repeat testings, and additional repeat experiments if any, by individual laboratories. For Labs 1 and 4, this amounts to 30 individual studies (28 drugs from initial testing and two drugs from blind repeat testings). For Lab 2, 36 individual studies were included (28 drugs from initial testing, two drugs from blind repeat testings, three additional ranolazine studies, and one additional study each for metoprolol, clozapine, and sotalol). For lab 3, 16 individual studies were done (14 drugs from initial testing, two drugs from blind repeat testings). For Lab 5, 33 studies were included (28 drugs from initial testing, two drugs from blind repeat testings, two additional metoprolol studies, and one additional astemizole study). Text labels in the plots show median (2.5th percentile, 97.5th percentile) of the SD of pIC_50_s. (**B**) pIC_50_ SD versus $\:{\text{p}\text{I}\text{C}}_{50,\:group\:avg}-{\text{p}\text{I}\text{C}}_{50,\:lab}$. A total of 145 experiments were conducted in this study and used in this analysis. r is the linear correlation coefficient.

At the level of individual drug experiments, the spread of fractional inhibition values from individual recordings across drug concentrations translates into uncertainty in block potency (and n_H_) for that experiment. Figure 7A shows cumulative distribution plots of the SD of pIC_50_s for individual laboratories across all drugs and all experiments (including blind repeat testings by all laboratories and additional repeat experiments by Labs 2 and 5). Collectively, Labs 3 and 4 have lower within-experiment variability in comparison with Labs 1, 2, and Lab 5. However, smaller within-experiment variability did not translate to that drug’s data being closer to the group average, as evidenced by the lack of relationship between pIC_50_ SD from individual experiments and $\:{\text{p}\text{I}\text{C}}_{50,\:group\:avg}-{\text{p}\text{I}\text{C}}_{50,\:lab}$ (Fig. 7B).

### Variability in hERG block potency

A goal of this study is to assess variability of hERG block potency, which is defined as the distribution of potencies for the same drug when measured repeatedly by the same laboratory. As this distribution was not empirically determined in the present study, two approaches—descriptive statistics and meta-analysis—were undertaken to provide estimates. These approaches used strategies to estimate and then remove drug- (i.e., potency) and laboratory-specific impacts (i.e., systematic differences to the group average) to reveal unexplained or residual variability in data not tied to drug or laboratory.

**Fig. 8:**
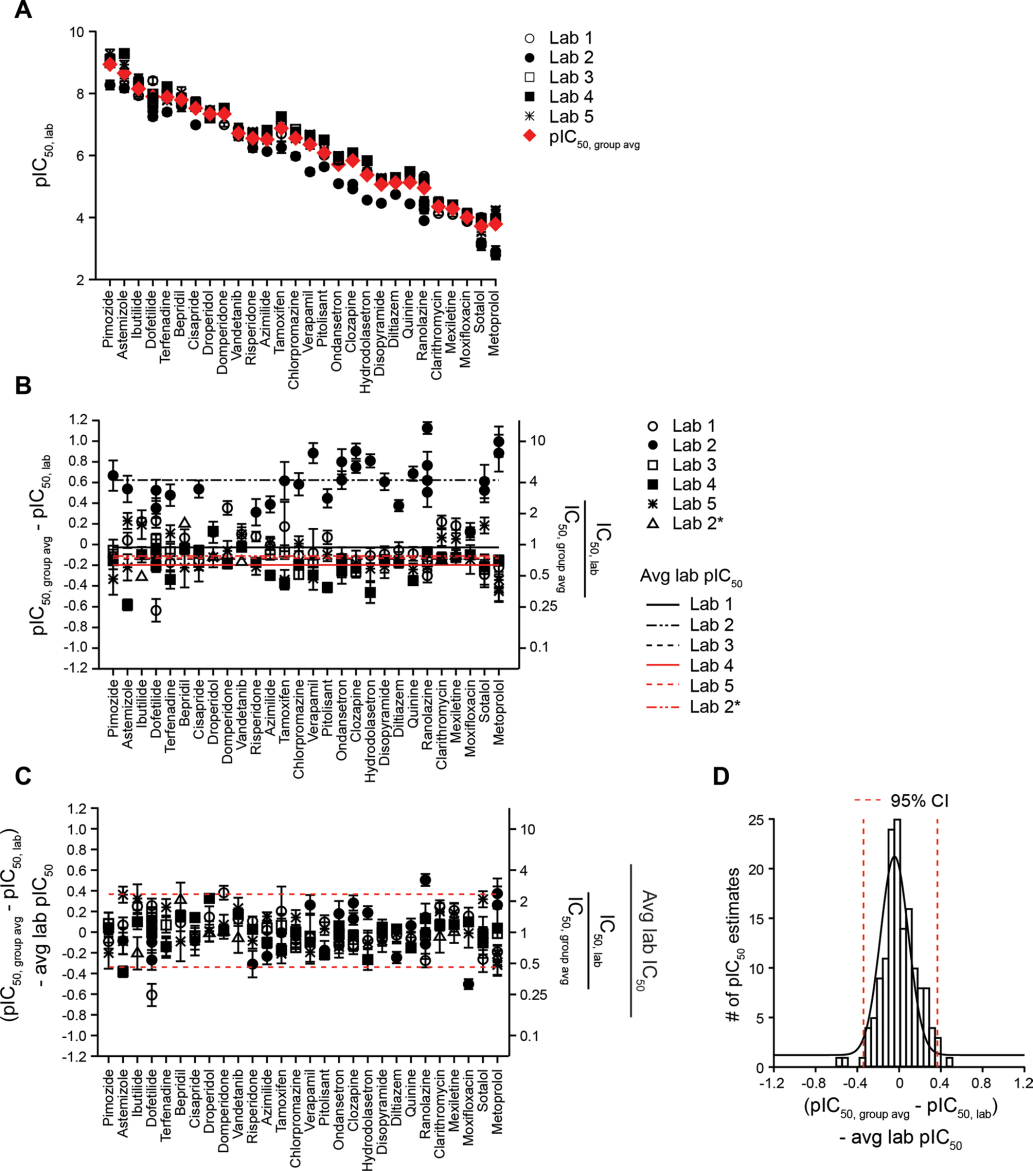
Descriptive statistics approach to estimating variability in hERG block potency. (**A**) Drugs shown in descending order of potencies. (**B**) To remove drug-specific effects, the average pIC_50_s for each drug ($\:{\text{p}\text{I}\text{C}}_{50,\:group\:avg}$) for all available data including repeat testings and additional repeat experiments (red diamonds) were subtracted from each individual pIC_50_ ($\:{\text{p}\text{I}\text{C}}_{50,\:lab}$). This is equal to $\:Log\left(\frac{{\text{I}\text{C}}_{50,\:lab}}{{\text{I}\text{C}}_{50,\:group\:avg}}\right)$ as shown in Figs. 1 and 4. After this subtraction step, laboratory-specific tendencies were estimated by averaging the $\:{\text{p}\text{I}\text{C}}_{50,\:group\:avg}-{\text{p}\text{I}\text{C}}_{50,\:lab}$ values for each lab. These averaged values are depicted here as horizontal lines for each lab. Lab 2 data for the last seven drugs studied (ibutilide, vandetanib, bepridil, clarithromycin, domperidone, droperidol, and mexiletine) were averaged separately (Lab 2*). The right Y-axis shows the equivalent changes in linear scale. (**C**) The laboratory-specific tendencies are removed, leading to center alignment of each laboratory’s remaining, unexplained values for each drug. (**D**) Histogram of the remaining, unexplained values shown in (**C**) to visualize normal distribution of the data (*N* = 145 studies). Dashed red lines are the 2.5th and 97.5th percentiles of the data (95% CI).

Figure 8 presents the descriptive statistics approach. Panel A shows the pIC_50_ values of the drugs in descending block potencies, and this drug order was used to present data in panels B and C. As in Figs. 1 and 4, the distance of each laboratory’s drug data to the group average was first calculated to remove drug-specific impact, and these distances were then averaged to derive laboratory-specific impacts, indicated by different lines for different laboratories in Fig. 8B. Lab 2’s systematic data differences to the other laboratories were not apparent for the last seven drugs it tested, hence data from Lab 2 were separated into groups with and without systematic differences, indicated as “Lab 2” and “Lab 2*” in Panels 8B and C, respectively, to derive two laboratory-specific impacts. Subtracting laboratory-specific impacts from $\:{\text{p}\text{I}\text{C}}_{50,\:group\:avg}-{\text{p}\text{I}\text{C}}_{50,\:lab}$ values for individual laboratories led to center alignment of all laboratories’ data, with the remaining values around the center considered as residual variability (Fig. 8C). Figure 8D shows the histogram of these remaining values. The difference between the 2.5th and 97.5th percentiles for these values was 0.7, the antilog of which is 5. Accordingly, the variability of hERG data obtained using this method is 5X, meaning that two IC_50_s less than 5X apart should not be considered as different.

**Fig. 9:**
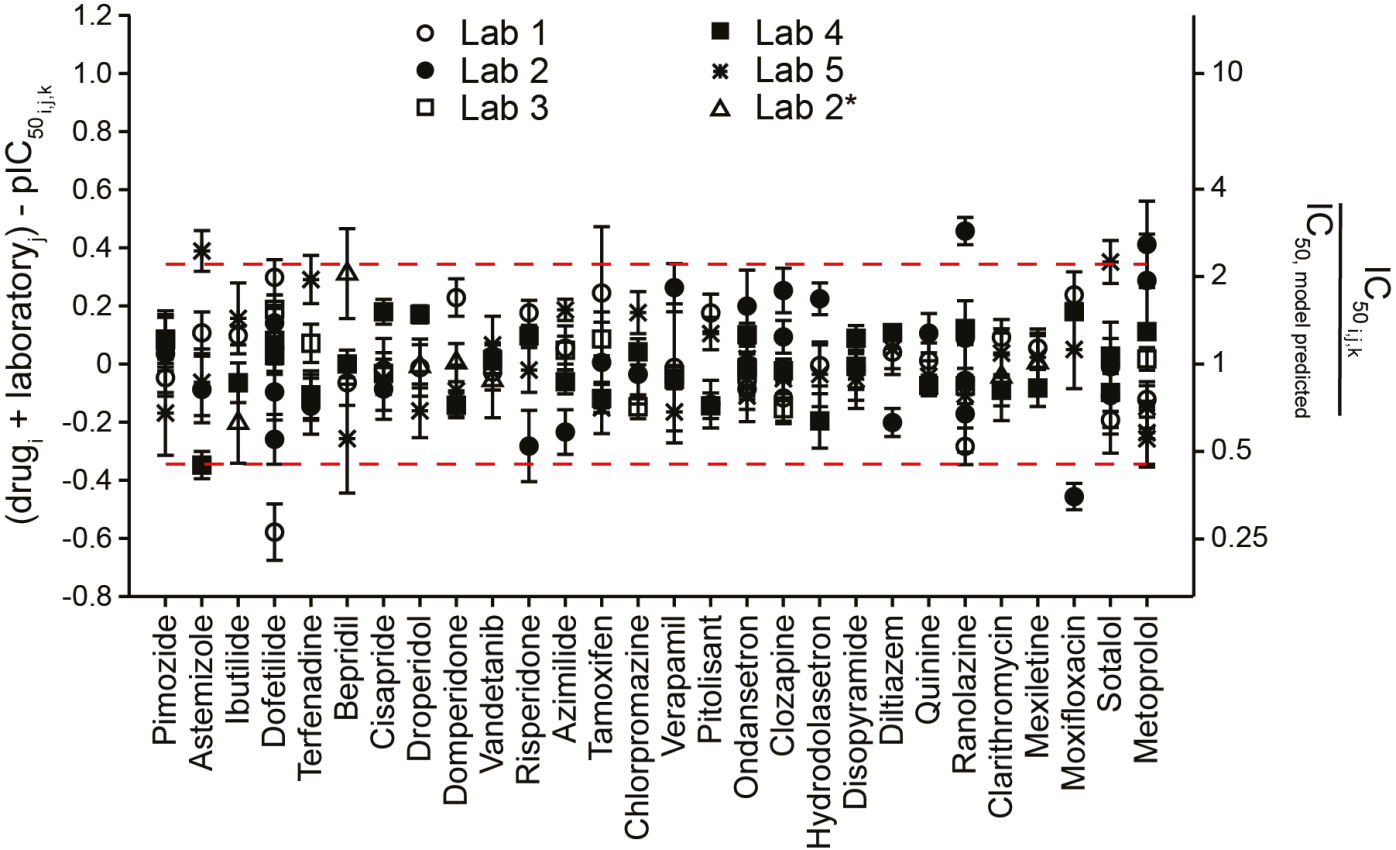
Modelling approach to estimating variability in hERG block potency. Plot of the variability between studies not explained by differences in drug potency and laboratory for all drugs, estimated using a mixed effects meta-analysis model. The left Y-axis shows the observed pIC_50_ for each drug from each laboratory ($\:pI{C}_{{50}_{i,j,k}}$) subtracted from the model-predicted pIC_50_ accounting for drug and laboratory effects ($\:dru{g}_{i}+laborator{y}_{j})$. The right Y-axis shows the equivalent changes in multiples relative to the model-predicted IC_50_. Dashed red lines represent the 95% CI.

The above method used the observed pIC_50_s in calculations and did not account for the within-experiment variability. Thus, a mixed effects meta-analysis approach was also used to estimate the residual variability in hERG block potency after accounting for systematic drug- and laboratory-specific impacts and the variability observed in individual experiments. Figure 9 shows the results of subtracting the observed pIC_50_s ($\:pI{C}_{{50}_{i,j,k}}$) from the model-estimated pIC_50_s that accounted for drug- and laboratory-specific impacts ($\:{drug}_{i}+{laboratory}_{j}$). The estimated overall variability (τ) not explained by drug and laboratory and expressed as SD was 0.18, and as 95% CI 0.69, corresponding to an IC_50_ ratio of 4.9X (95% CI: 4.0 to 6.2). This measure is closely aligned with the descriptive statistics approach above.

**Fig. 10:**
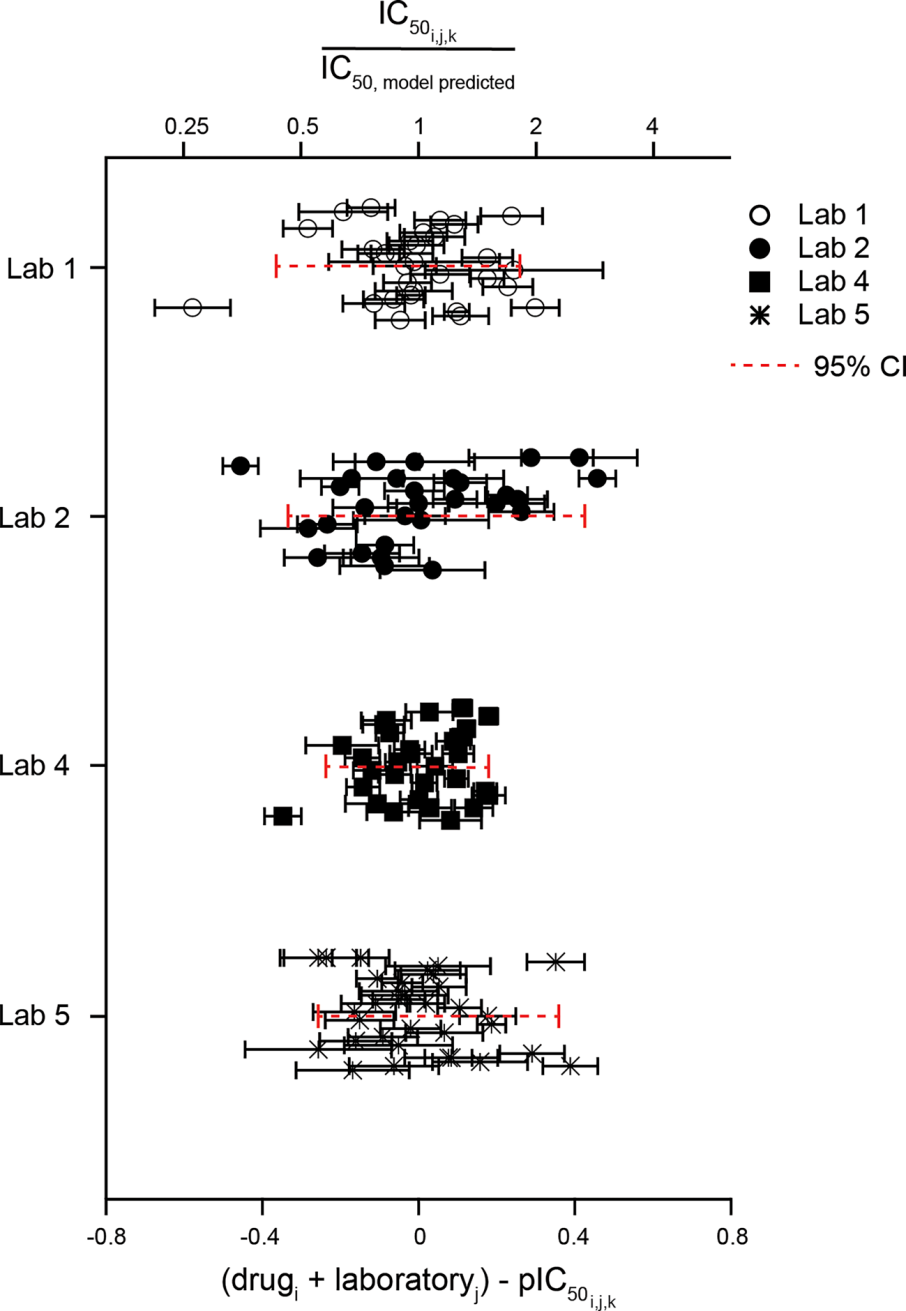
Laboratory-specific variability estimated using the location-scale meta-analysis model. Plot of variability in hERG block potencies for each laboratory that participated in Phase 1 and Phase 2. For Lab 2, only Phase 1 drugs and the first seven drugs studied during Phase 2 are included. The bottom X-axis shows the pIC_50_ for each drug from each laboratory ($\:pI{C}_{{50}_{i,j,k}}$) subtracted from the model-predicted pIC_50_ accounting for drug and laboratory effects ($\:dru{g}_{i}+laborator{y}_{j})$. The top X-axis shows the equivalent changes in multiples relative to the model-predicted IC_50_. Model estimations included all laboratories and drugs in the study and any repeats by each Lab. Dashed red lines represent the 95% CI.

The mixed effects model used to estimate the overall variability assumes that the residual variability is homogeneous across different drugs and laboratories. To explore if the residual variability differs by laboratory, a location-scale model was applied using laboratory as a predictor of the residual variability (Fig. 10). These residual variabilities expressed as IC_50_ ratios are as follows: Lab 1, 5.3 (3.4 to 9.7), Lab 2 during Phase 1 and the first half of Phase 2, 9.6 (5.4 to 20.8), Lab 4, 3.4 (2.3 to 6.0) and Lab 5, 4.7 (3.1 to 8.6). An estimate was not done for Lab 3 owing to its lack of Phase 2 data.

## Discussion

During drug development, the hERG results are an integral part of cardiac safety evaluation to support first-in-human studies. Recent updates to ICH E14 Q&As created new pathways for using negative hERG findings in the nonclinical-clinical integrated risk assessment to rule out the likelihood of clinical QT_C_ prolongation and associated proarrhythmia—a conclusion that previously relied on clinical findings only. This new use of the hERG results has prompted questions regarding what safety margin to use and how to interpret the hERG results. Derivation of a new hERG safety margin requires both high quality patch clamp and ECG data for many drugs—out of scope for the present study. Here the focus is on making the patch clamp data publicly available (https://osf.io/a6k5t/) to enable derivation of a new hERG safety margin, and importantly achieve a better understanding of different types of data variabilities—across laboratories, within an experiment, and for the hERG assay collectively—knowledge necessary to conclude whether an investigational product (IP) is like or unlike the reference torsadogens. Using hERG block potencies of 28 drugs generated by five laboratories using the manual patch clamp method, a standardized protocol, and following ICH S7B Q&A 2.1 best practices, the central findings are:

1. Systematic differences in hERG data across laboratories can still occur. These differences are not explained by drug exposure, pharmacological sensitivity of the cell lines, and data quality.
2. HERG block potencies for the same drug by the same laboratory measured on different occasions can show substantial variability (i.e., much larger than within-experiment variability).
3. Variability in hERG block potency, based on pooling data across all drugs and laboratories, is ~ 5X.

Systematically different data across laboratories, especially after exposure and data quality are ascertained, highlight the inherent difficulty of the “one safety margin” approach to define a negative assay. In ICH S7B Q&A 2.1, there is a statement that reads “if positive control data fall outside the range of expected values, then the study is inconclusive”, but that range is not defined. In light of the findings outlined above, the definition of “range of expected values” also warrants revisiting. Some ideas are presented below, with the intent of fostering discussions amongst stakeholders to continue developing a data interpretation scheme based on real world evidence, and in doing so bolstering confidence for using nonclinical results in regulatory decision-making.

### Across laboratory data variability

That hERG block potencies generated using standardized protocols can show systematic differences across laboratories was previously reported by Hanson et al.^14^. In that study, two laboratories measured hERG block potencies for 12 drugs using standardized voltage waveform, stimulation frequency, recording solutions, and blinded drug stocks. Drug exposures were determined and corrected for nine out of the 12 drugs. Of the nine drugs for which IC_50_ values could be established, one laboratory showed higher values for eight drugs, with differences ranging from 3X to 40X. The potential source(s) of these systematic data differences including data quality were not explored. The present work confirms and extends findings by Hanson et al., showing that hERG block potencies across laboratories can be systematically different after protocol standardization, following ICH S7B Q&A 2.1 best practices which includes concentration verification (Figs. 1,  4, and 8B), and supported by independent data quality verification (Fig. 6; Supplemental Fig. 6; for reports of current traces and time course plots for every cell: https://osf.io/a6k5t/). Systematic differences were not due to higher amounts of drug loss (Figs. 1,  4, and 5), method of drug application (Fig. 2A), drug sources and stocks (Fig. 2A and C), data quality (Fig. 6 and Supplemental Fig. 6), or pharmacological sensitivity of the cell lines (Fig. 2B). Given that the source(s) underlying systematic data differences were not identified, no further protocol standardization is recommended at this time.

One recording procedure that was not standardized across laboratories in this study was the degree of series resistance compensation. Thus, Lab 3 did not compensate; Labs 1, 2, 4 and 5 compensated at 50 to 80%. Series resistance causes voltage drop, leading to deviation of the membrane potential from the command potential. Therefore, in whole cell voltage clamp experiments, series resistance can result in distortion of the current-voltage relationship for voltage-gated ion channels, and for fast and large currents such as those mediated by Ca_V_1.2 and Na_V_1.5 channels, lead to peak current attenuation and kinetic distortion. For hERG current elicited by the repolarizing voltage ramp, turning off series resistance compensation in the middle of the recording can lead to a shift in the ramp voltage, reflecting voltage error and current-voltage distortion, without changing the current amplitude. This is due to the fact that the membrane voltage that elicits the maximal number of open hERG channels was eventually reached, and that the hERG current amplitude is a function of the number of open channels and driving force. Since most hERG blockers including ones tested in this study are open channel blockers, that the maximal number of open channels during the voltage ramp is not impacted by compensation suggests that series resistance unlikely explains the systematic data differences found in this study.

### Variability in hERG block potency

Repeat testings of dofetilide and ondansetron in a blind fashion yielded hERG block potencies within 1.6X of the initial testing results, except for data from one laboratory for which the drug potency for the dofetilide repeat was 7.6X higher than its initial testing result (Fig. 3). These results demonstrate that hERG block potencies for the same drugs generated by the same laboratory on separate occasions can show variability beyond that approximated in a single experiment. The limited repeat experiments conducted here were simply insufficient to establish the full potency distribution even for a single drug. Thus, alternative approaches utilizing descriptive statistics and meta-analysis were pursued. Both approaches showed that variability of the hERG block potencies is ~ 5X (Figs. 8 and 9), even though meta-analysis accounted for within-experiment variability while descriptive statistics did not, indicating little impact of this extra step based on the data in this study. This finding is consistent with results presented in Fig. 7, demonstrating no relationship between data variability in an experiment and proximity of pIC_50_ from that experiment to the center of distribution. Similar results were also presented by Leishman et al.^22 ^suggesting that in general, between-experiment variability dwarfs within-experiment variability. Leishman et al., studied variability in hERG data across laboratories for three drugs. Information regarding the voltage protocol(s) and pacing rate(s) used was not provided, and drug concentrations were measured for only two of the six laboratories in the study. No systematic data differences were found across participating laboratories. Using the plasma concentration and percent protein binding values from the ICH E14/S7B training materials (https://database.ich.org/sites/default/files/ICH_E14-S7B_TrainingMaterial_ExamplesSupplementalFile_2022_0331.pdf), the study showed that ondansetron’s safety margin is distinct from those of dofetilide and moxifloxacin^22^. Whether ondansetron is a typical hERG blocker or not remains unclear. Results from the present study can be used with ECG data for additional hERG blockers to address the distribution of hERG safety margins.

### Regulatory implications

The present study as well as Hanson et al.^14^ underscores the challenge of using one hERG safety margin threshold to identify IPs with a likelihood for hERG block-mediated QT_C_ prolongation. Systematic data differences can emerge and resolve, and a survey with participating laboratories in this study indicates that some, in addition to Lab 2, have experienced this phenomenon first-hand, or are aware of its occurrence. Thus, some ideas regarding when and how to consider the possibility of systematic data differences as well as implications of variability in hERG block potency are shared below. For simplicity sake, these scenarios assume that a reference hERG safety margin threshold for nonclinical-clinical integrated risk assessment has been presented in the literature, based on high quality patch clamp data generated following best practices and clinical ECG data for many “reference drugs” (i.e., predominant hERG blockers with known TdP risk; https://database.ich.org/sites/default/files/ICH_E14-S7B_TrainingMaterial_ExamplesSupplementalFile_2022_0331.pdf).

The hERG assay performed to support cardiac safety assessment typically includes data for an IP and a positive control, which is one of the reference drugs used to establish the reference margin threshold. For a laboratory to compare its IP’s safety margin to the reference margin threshold, in principle the laboratory should demonstrate that its hERG block potency of the positive control, measured using the same experimental protocols and following best practices, is similar to the data used to derive the reference margin threshold. To determine whether two hERG block potency values are similar or not, variability in hERG block potency as defined in this study should be accounted for. The impact of a difference between the hERG data for the positive control versus the reference likely depends on the distance between the IP’s safety margin relative to the reference margin threshold. In other words, as the IP’s safety margin moves closer to the reference margin threshold, the need to understand potential systematic difference in the hERG results for a laboratory increases.

### Limitations and lessons learned

Firstly, variability in hERG block potency was not empirically determined. Rather, this measure was approximated by pooling data across laboratories and drugs then using two approaches—descriptive statistics or meta-analysis—to remove drug- and laboratory-specific impacts to assess the distribution of the remaining, unexplained values. Secondly, for both approaches, all drugs are assumed to have the same variability in hERG block potency. However, based on their expertise in ion channel pharmacology, participating laboratories unanimously asserted that this variability should be different for different drugs, with tighter distributions for drugs with faster block kinetics and no instability issues under the experimental conditions. The modelling results shown in Fig. 10 also suggest that variability in hERG block potency is laboratory-specific, ranging from 3.4X to 9.6X for different laboratories in this study. Nonetheless, in the absence of empirical data demonstrating distributions of hERG block potencies for the same drug obtained repeatedly, participating laboratories also agreed that ~ 5X is not unreasonable. Thus, when interpreting the hERG results from a laboratory that has not defined its own variability in hERG block potency, the 5X reported in this study could be imposed (i.e., ± 2.5X around an IC_50_ estimate) to define the range within which the hERG data should not be considered different.

Empirically testing the same drug repeatedly to understand variability in hERG block potency using the manual patch clamp method is labor-intensive and slow. Depending on the characteristics of the drug (i.e., stability and block kinetics) and cell line at the time of testing, data collection to generate a concentration-inhibition curve (i.e., one experiment) can take from a day to a week. Here, the automated patch clamp approach would offer significant advantages over the manual patch clamp method. Variability in hERG block potencies for automated patch clamp data, similar to those presented in the literature^23–25 ^could be estimated empirically for the positive control, IP, and additional drugs as needed concomitantly, bypassing the need of using literature variability measures and demonstrating the presence or lack of systematic data variability for data interpretation.

Variability in drug potency applies to all ion channel studies. The same laboratories that generated data for this study are continuing to assess drug inhibition of Ca_V_1.2 and peak and late Na_V_1.5 currents to promote better understanding of variability and create a new ion channel database to support future modelling research for proarrhythmia risk prediction (https://hesiglobal.org/event/safety-pharmacology-society-annual-meeting-2024/).

## Conclusion

The new ICH E14/S7B Q&As describe regulatory pathways for using negative nonclinical data, including hERG, to complement clinical QT_C_ data for risk interpretation. A fundamental knowledge gap that creates challenges in implementing these pathways is a lack of hERG data generated following ICH S7B Q&A 2.1 best practices that captured real world data variability—both across and within laboratories—for as many drugs with diverse physicochemical properties as possible. Such a dataset is needed for two reasons: (1) to assess and integrate hERG safety margins for many clinical drugs that block hERG channels, are associated with TdP risk, and have high quality clinical ECG data to yield a margin threshold; and (2) to understand just how different two hERG block potency values, and by extension safety margin values, need to be for these to be interpreted as different. The present study filled this knowledge gap, demonstrating that systematic and reproducible data differences across laboratories could occur and raise a need to develop laboratory-specific hERG assay interpretation method. Variability of hERG block potencies as presented is neither drug- nor laboratory-specific, hence provides a reasonable estimate to use when interpreting data from laboratories that have yet to define their data reproducibility. The dataset is fully available to lend credibility to the study conclusions and support additional cardiac safety research including margin threshold derivation.

## Supplementary Information

Below is the link to the electronic supplementary material.

Supplementary Material 1

Supplementary Material 2

Supplementary Material 3

Supplementary Material 4

Supplementary Material 5

Supplementary Material 6

Supplementary Material 7

Supplementary Material 8

Supplementary Material 9

Supplementary Material 10

## Data Availability

All data used to generate this manuscript, including original electrophysiology source waveforms, are available at https://osf.io/a6k5t/.
