# Supplementary material for: Multi-laboratory comparisons of manual patch clamp hERG data generated using standardized protocols and following ICH S7B Q&A 2.1 best practices
Source: Sci Rep. 2025 Aug 16;15:29995. doi: 10.1038/s41598-025-15761-8 (PMC12357877; doi:10.1038/s41598-025-15761-8)
Supplement: Supplementary file 2 — Supplementary Material 2 [file 41598_2025_15761_MOESM2_ESM.docx]

**Supplemental materials for:**

**Multi-laboratory comparisons of manual patch clamp hERG data generated using standardized protocols and following ICH S7B Q&A 2.1 best practices**

Claudia Alvarez Baron^1^, Jun Zhao^1^, Huimei Yu^1,2^, Ming Ren^1^, Nicolas Thiebaud^1,3^, Donglin Guo^4^, Giri Vegesna^1^, Cheng-Hui Hsiao^1^, Ryan DePalma^1^, Sabyasachy Mistry^1^, Isra Tariq^1^, Md Shadiqur Rashid Roni^1^, Omnia A. Ismaiel^1,5^, Murali K. Matta^1,6^, Vikram Patel^1^, Manni Mashaee^1^, Jose Vicente^4,7^, Lars Johannesen^4^, Jiansong Sheng^8^, Simon Hebeisen^9^, James Kramer^10^, Andrew Bruening-Wright^10^, Koji Nakano^11^, Hiroshi Matsukawa^11^, Jennifer Beck Pierson^12^, Wendy W. Wu^1^*

^1^Division of Applied Regulatory Science, Office of Clinical Pharmacology, Center for Drug Evaluation and Research, US Food and Drug Administration, Silver Spring, MD, USA. ^2^Division of Pharmacology/Toxicology Review, Office of Safety and Clinical Evaluation, Office of Generic Drugs, Center for Drug Evaluation and Research, US Food and Drug Administration, Silver Spring, MD, USA. ^3^Vertex Pharmaceuticals (Europe) Ltd, Abingdon, Oxfordshire, UK. ^4^Division of Cardiology and Nephrology, Office of Cardiology, Hematology, Endocrinology and Nephrology, Office of New Drugs, Center for Drug Evaluation and Research, US Food and Drug Administration, Silver Spring, MD, USA. ^5^Division of Nonclinical Science, Office of Science, Center for Tobacco Products, US Food and Drug Administration, Silver Spring, MD, USA. ^6^Department of Pharmacokinetics, Dynamics, Metabolism, and Bioanalysis, Merck & Co., Inc., West Point, PA, USA. ^7^Translational Cures, Washington, DC, USA. ^8^CiPA Lab, Gaithersburg, MD, USA. ^9^B’SYS GmbH, Witterswil, Switzerland. ^10^Charles River Laboratories, Cleveland, OH, USA. ^11^Drug Safety Testing Center Co., Ltd., Higashimatsuyama Laboratories, Saitama, Japan. ^12^Health and Environmental Science Institute, Washington, DC, USA.

^2,3,5,6,7^Current affiliation

# **Supplemental Figure Legends**

**Supplemental Figure 1.** *hERG current traces from representative cells recorded by Labs 1 and 2 and time course plots of ramp current amplitude, I_-80 mV_, and R_input_.* **A)** The voltage waveform used in this study (see method section on *“Electrophysiology and drug application”* for details). **B)** *Top left,* Current traces recorded by Lab 1 in control solution (black), following applications of 0.01 (dark gray) then 0.03 µM dofetilide (light gray), and then 1 µM E-4031 (red) to eliminate residual hERG current. Traces show the last recorded current in each solution. *Bottom,* voltage waveform. *Top right,* Current traces after subtracting the average of the last five traces in E-4031. Scale applies to not subtracted and subtracted traces. **C)** Time course plots of the ramp current amplitude, I_-80 mV_, and R_input_ for the cell in **(B)**. **D)** *Top left,* Current traces recorded by Lab 2 in control solution (black), following applications of 30 µM hydrodolasetron (dark gray), and then 1 µM E-4031 (red). Traces show the last recorded current in each solution. *Top right,* Current traces after subtracting the average of the last five traces in E-4031. Scale applies to not subtracted and subtracted traces. **E)** Time course plots of the ramp current amplitude, I_-80 mV_, and R_input_ for the cell in **(D)**.

**Supplemental Figure 2.** *hERG current traces from representative cells recorded by Labs 3, 4 and 5 and time course plots of ramp current amplitude, I_-80 mV_, and R_input_.* **A)** *Top left,* Current traces recorded by Lab 3 in control solution (black), following applications of 3 µM ondansetron (dark gray), and then 0.5 µM E-4031 (red) to eliminate residual hERG current. Traces show the last recorded current in each solution. *Bottom,* voltage waveform. *Top right,* Current traces after subtracting the average of the last five traces in E-4031. Scale applies to not subtracted and subtracted traces. **B)** Time course plots of the ramp current amplitude, I_-80 mV_, and R_input_ for the cell in **(B)**. **C)** *Top left,* Current traces recorded by Lab 4 in control solution (black), following applications of 2 (dark gray) then 8 µM disopyramide (light gray), and then 1 µM E-4031 (red). Traces show the last recorded current in each solution. *Top right,* Current traces after subtracting the average of the last five traces in E-4031. Scale applies to not subtracted and subtracted traces. **D)** Time course plots of the ramp current amplitude, I_-80 mV_, and R_input_ for the cell in **(C)**. **E)** *Top left,* Current traces recorded by Lab 5 in control solution (black), following applications of 1 (dark gray) then 10 µM pitolisant (light gray), and then 1 µM E-4031 (red). Traces show the last recorded current in each solution. *Top right,* Current traces after subtracting the average of the last ten traces in E-4031. Scale applies to not subtracted and subtracted traces. **F)** Time course plots of the ramp current amplitude, I_-80 mV_, and R_input_ for the cell in **(E)**.

**Supplemental Figure 3.** *Simulated data illustrating within-experiment variability and variability in hERG block potency.* Panels **(A)** to **(C)** show three sets of simulated experiments with potencies drawn from the distribution shown in panels **(D)** as pIC_50_ and **(E)** as IC_50_ (nM). Between-experiment variability is set at ~0.06. In each panel, the IC_50_ and n_H_ along with their respective lower and upper 95% CI are shown on the top. Median IC_50_ was 13.50 nM with a ratio of the 97.5^th^ (33.32 nM) and 2.5^th^ (5.47 nM) percentiles of 6.09. This corresponds to a mean pIC_50_ of 7.87 with a standard deviation of 0.20.

**Supplemental Figure 4.** *Concentration-inhibition plots for verapamil.* These plots were generated using Lab 3’s data. Open symbols reflect individual data points. The data points were fit with the Hill equation to yield IC_50_, n_H_, and their respective 95% CI for **(A)** and **(B)**, and -pIC_50_ and its 95% CI for **(C)**. The solid gray curve illustrates the fit; the dotted gray curves illustrate the upper and lower bands of 95% CI for the fit. Values presented are parameter ± 95% CI. **A)** Fractional inhibition was plotted against nominal drug concentrations. **B)** Fractional inhibition was plotted against the corrected drug concentrations to account for loss due to nonspecific binding. **C)** Corrected drug concentrations were expressed as the common log of concentration in molar. The pIC_50_ values and their respective 95% CI are used to generate Figure 2. Note that in panels **(B)** and **(C)**, data for the highest concentration tested in patch clamp study (i.e., 10 µM in panel **(A)**) are not shown because drug solutions were not available for that concentration for concentration verification.

**Supplemental Figure 5.** *IC_50_ values for individual Phase 1 drugs from different laboratories.* The IC_50_ values were calculated by fitting the concentration-inhibition graphs for individual drugs from individual laboratories using the fractional inhibitions reported by each lab. Drug concentrations were corrected by accounting for percent losses measured from drug samples collected in satellite experiments. Error bars (± 95% CI) are present and obscured by the symbols.

**Supplemental Figure 6.** *Histogram plots of I_-80 mV_, R_input_, and hERG current characteristics from all recorded cells during vehicle control solution.* I_-80 mV_ is the current necessary to alter the membrane voltage from the cell’s resting membrane potential to -80 mV. R_input_ is the resting input resistance calculated using the pulse from -80 mV to -90 mV at the beginning of the voltage waveform. These two parameters are reflective of activities of ion channels that set the resting membrane potential and the artificial leak current resulting from the contact between the membrane and glass pipette tip. The maximum amplitude of hERG current during the voltage ramp down phase informs the expression level of cells used by individual laboratories. The voltage at which hERG current peaked during the voltage ramp down phase of the voltage waveform informs voltage-dependence of hERG current gating, and in cases of large current amplitudes, potential voltage errors. Ramp voltages are corrected for the liquid junction potential resulting from the recording solutions used. For each cell, these measurements were calculated using the average from the last four to five traces recorded in the vehicle solution, just prior to drug application. Each row represents data from one laboratory. The number below each laboratory’s name indicates the total number of cells recorded. For the histograms, the Y-axis reflects cell count; the X-axis reflects values of the parameter shown above each column. The values in the plots indicate mean ± SE.

**Supplemental Figure 7.** *IC_50_ values for individual Phase 2 drugs from different laboratories.* The IC_50_ values were calculated by fitting the concentration-inhibition graphs for individual drugs from individual laboratories using the fractional inhibitions reported by each lab. Drug concentrations were corrected using satellite samples for Labs 1, 4 and 5 and real samples for Lab 2. Panel. The first seven drugs tested by Lab 2 are indicated by an asterisk (*). Error bars (± 95% CI) are present and obscured by the symbols.

# **Supplemental Tables**

**Supplemental Table 1.** Sources of individual drugs used by the participating laboratories.

**Supplemental Table 2.** Survey of cell line and drug handling procedures.

**Supplemental Table 3.** Concentration-dependent drug loss from specific laboratories.

**Supplemental Table 4.** pIC_50_ and 95% CI estimated using corrected drug concentrations.

**Supplemental Table 5.** IC_50_s and n_H_ estimated using nominal drug concentrations.

**Supplemental Table 6.** IC_50_ and n_H_ estimated using corrected drug concentrations.

**Supplemental Table 7.** Thermal stability of metoprolol, verapamil, and clozapine in hERG external solution at 37°C.

**Supplemental Table 8.** Initial testing and subsequent testing(s) for select drugs.

**Supplemental Table 9.** pK_a_, LogP, and mean percent losses for individual drugs seen in satellite or real samples.

**Supplemental Table 10.** Linearity ranges established for LC-MS/MS bioanalysis.

**Supplemental Table 11.** Concentrations measured in bioanalysis samples and percent drug losses for each drug and laboratory.
