# Supplementary figures and images for: Multi-laboratory comparisons of manual patch clamp hERG data generated using standardized protocols and following ICH S7B Q&A 2.1 best practices
Source: Sci Rep. 2025 Aug 16;15:29995. doi: 10.1038/s41598-025-15761-8 (PMC12357877; doi:10.1038/s41598-025-15761-8)

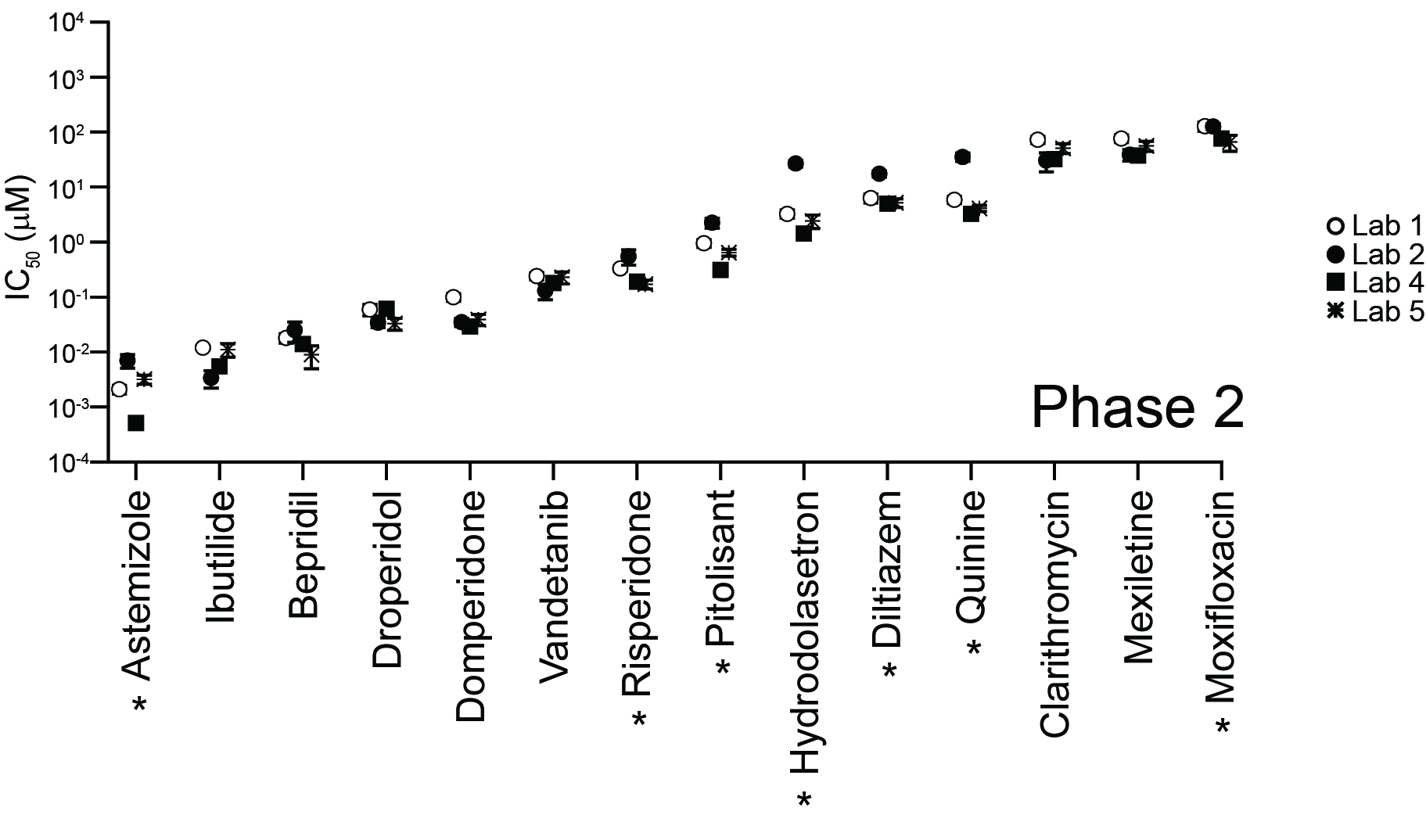

Supplement: Supplementary file 4 — Supplementary Material 4 [file 41598_2025_15761_MOESM4_ESM.tif]

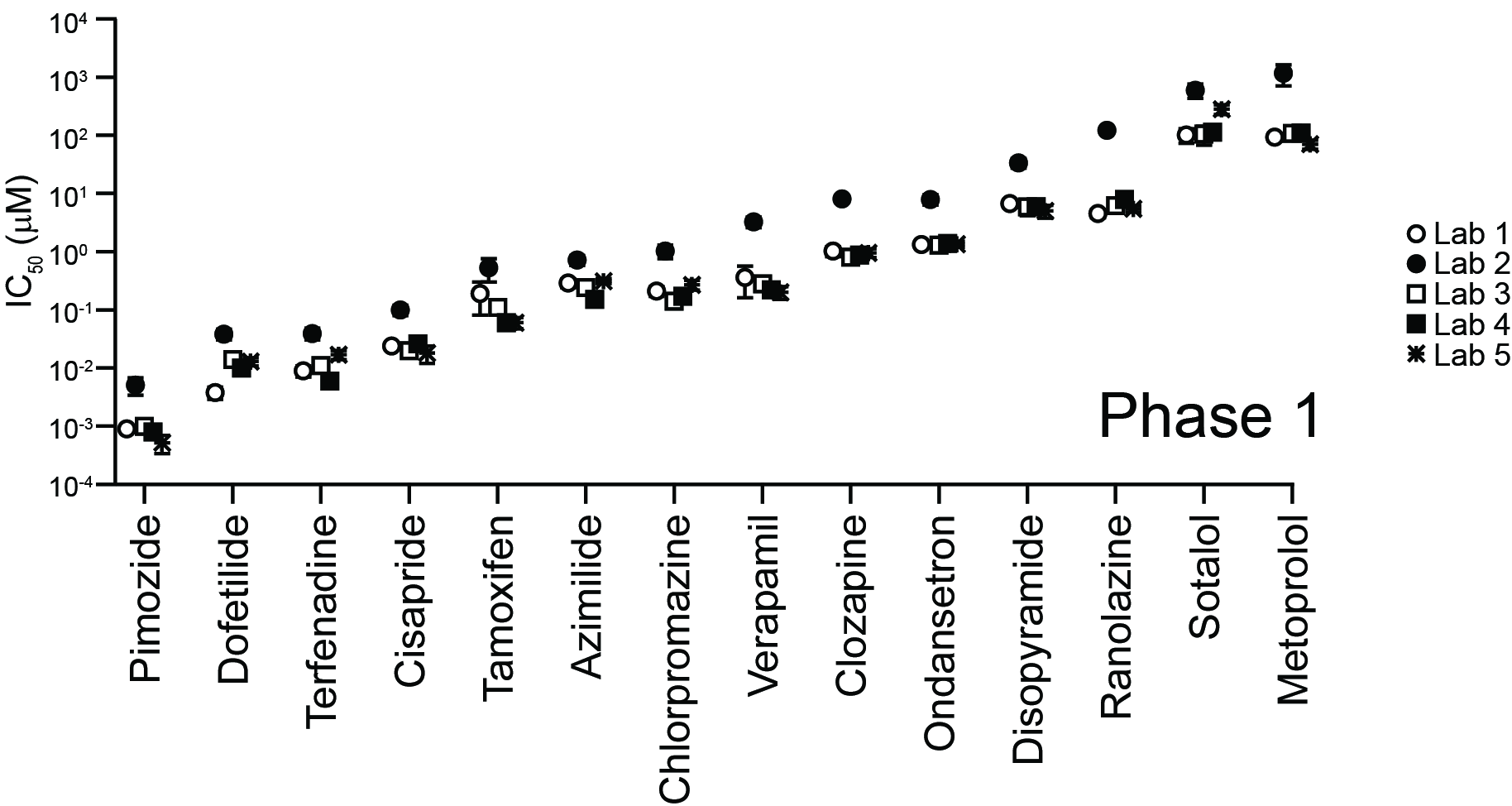

Supplement: Supplementary file 5 — Supplementary Material 5 [file 41598_2025_15761_MOESM5_ESM.tif]

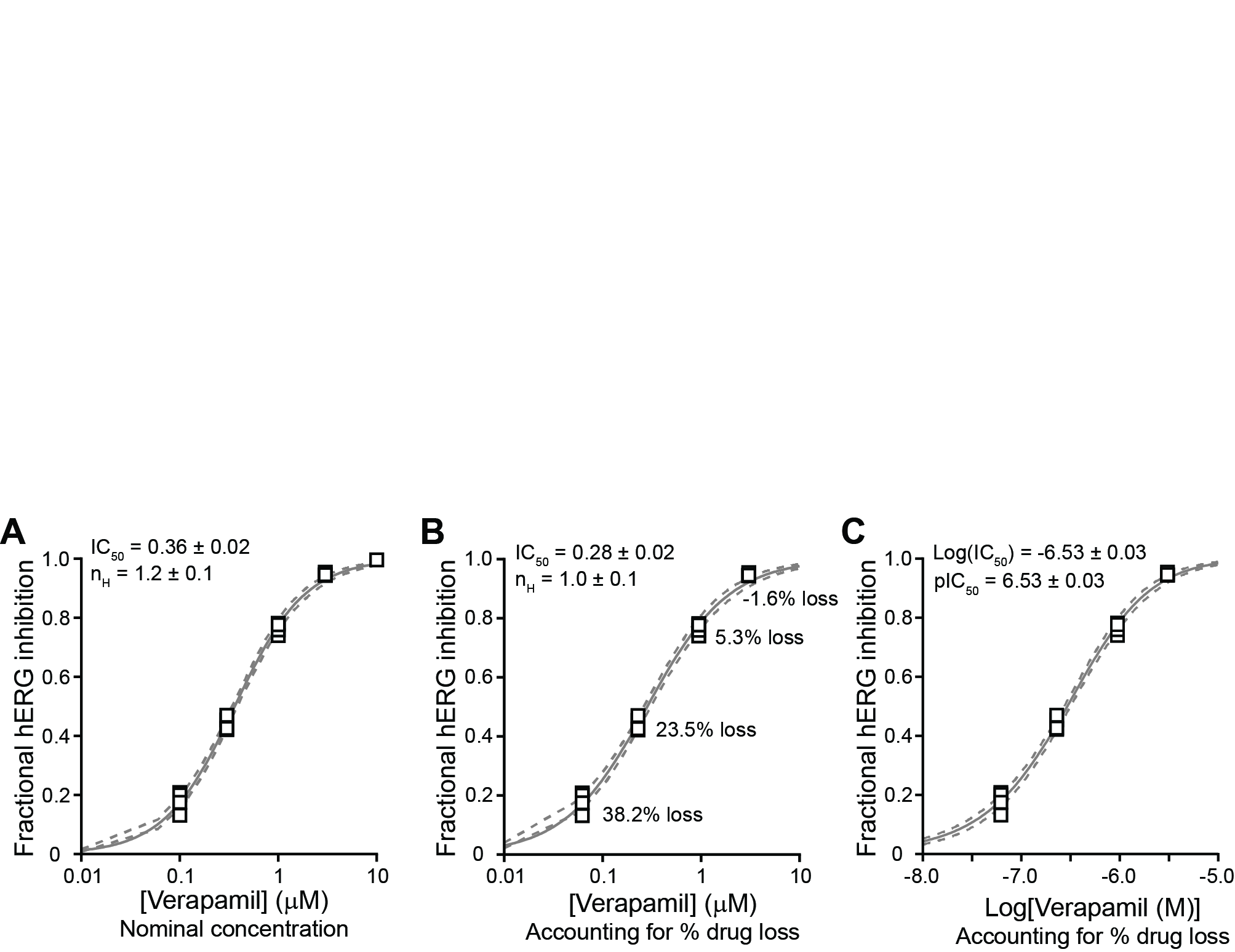

Supplement: Supplementary file 6 — Supplementary Material 6 [file 41598_2025_15761_MOESM6_ESM.tif]

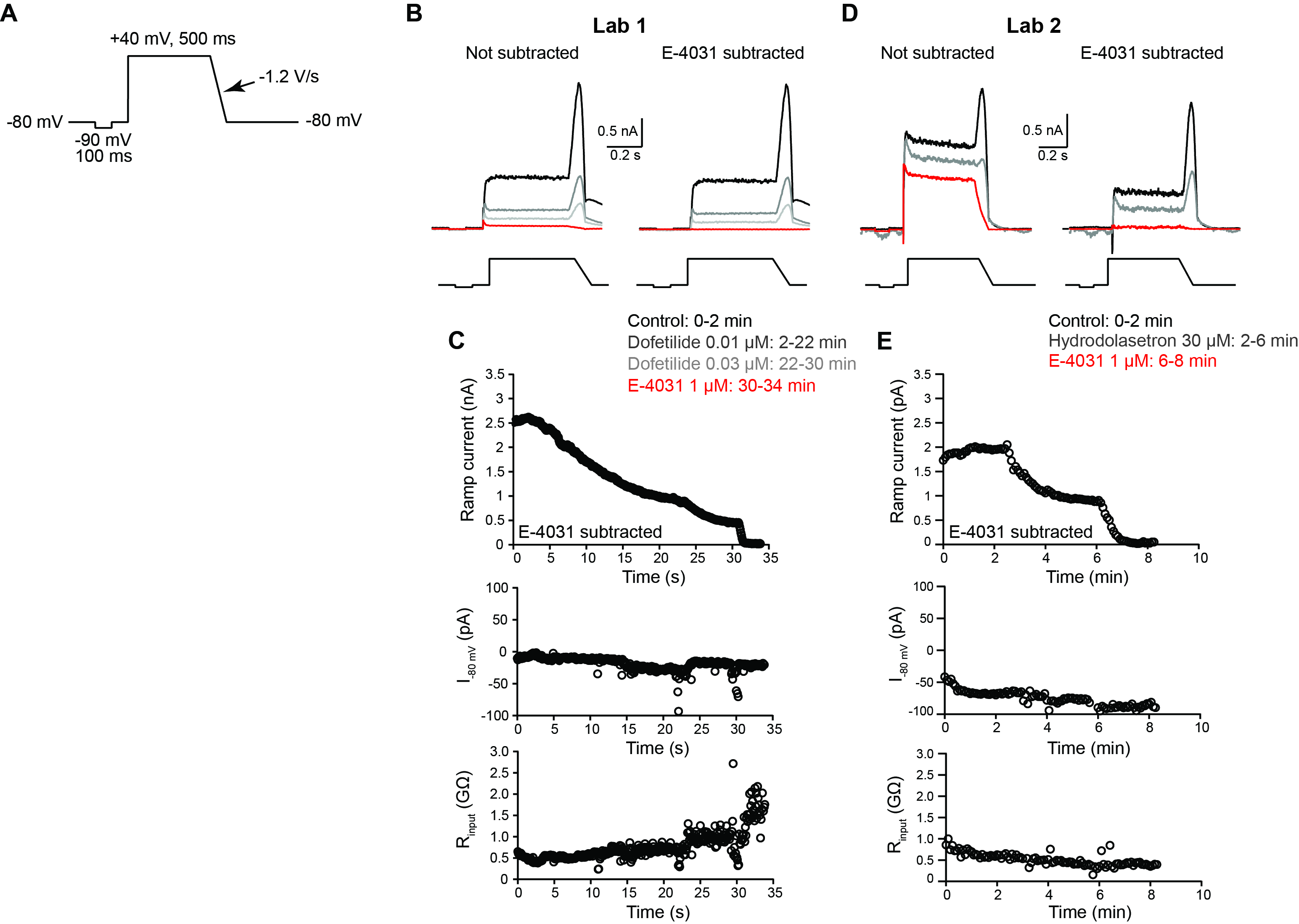

Supplement: Supplementary file 7 — Supplementary Material 7 [file 41598_2025_15761_MOESM7_ESM.tif]

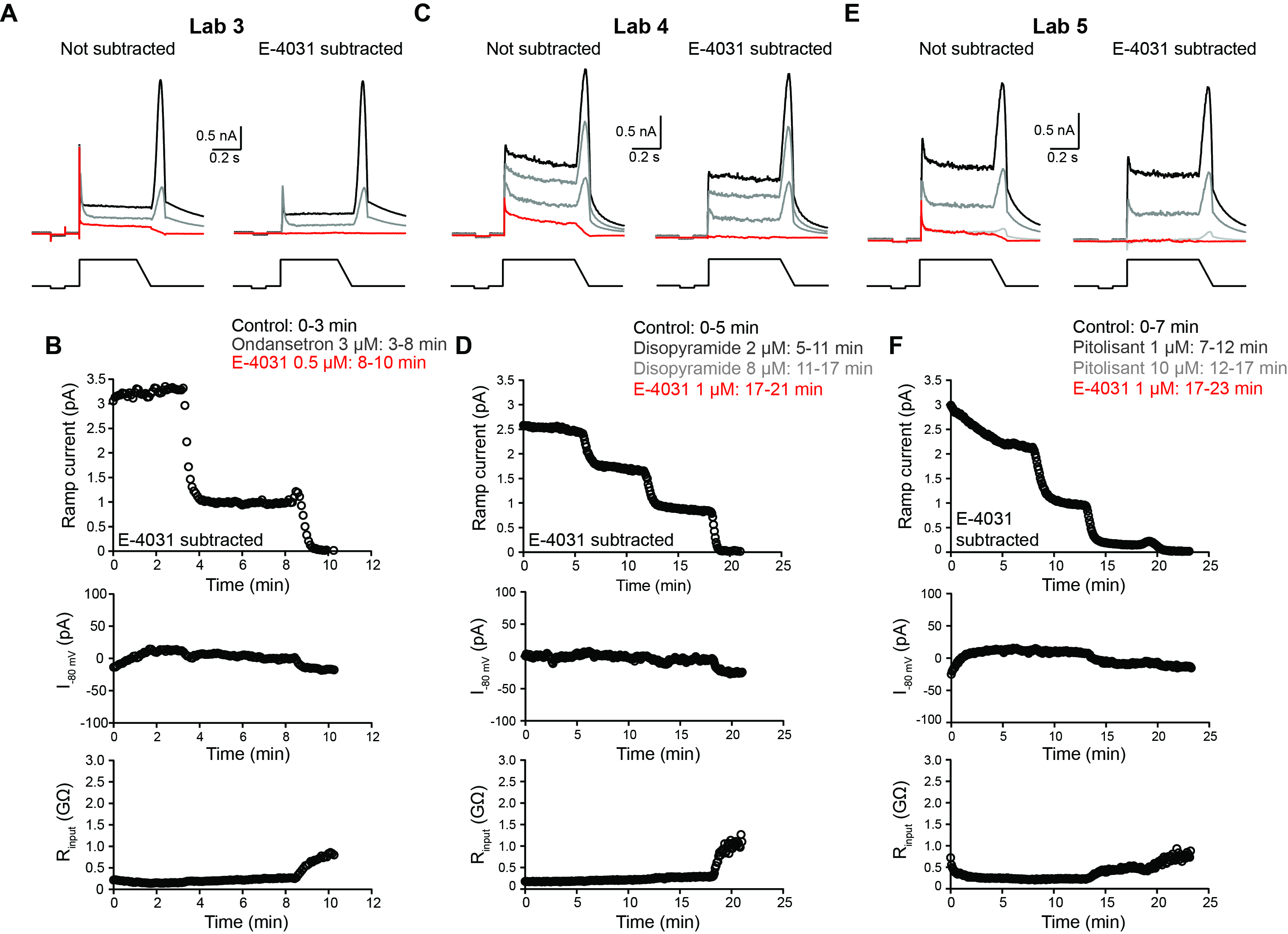

Supplement: Supplementary file 8 — Supplementary Material 8 [file 41598_2025_15761_MOESM8_ESM.tif]

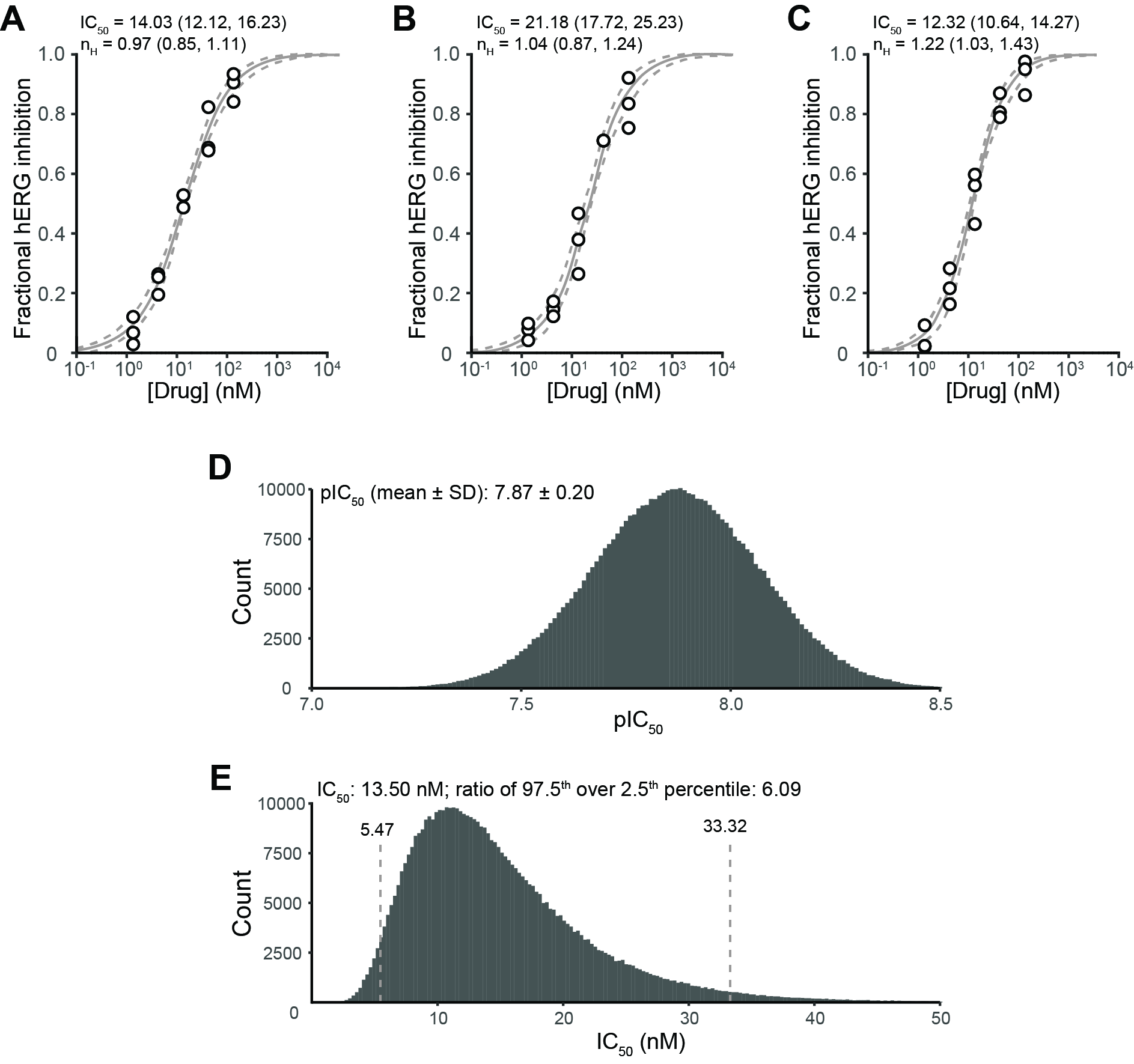

Supplement: Supplementary file 9 — Supplementary Material 9 [file 41598_2025_15761_MOESM9_ESM.tif]

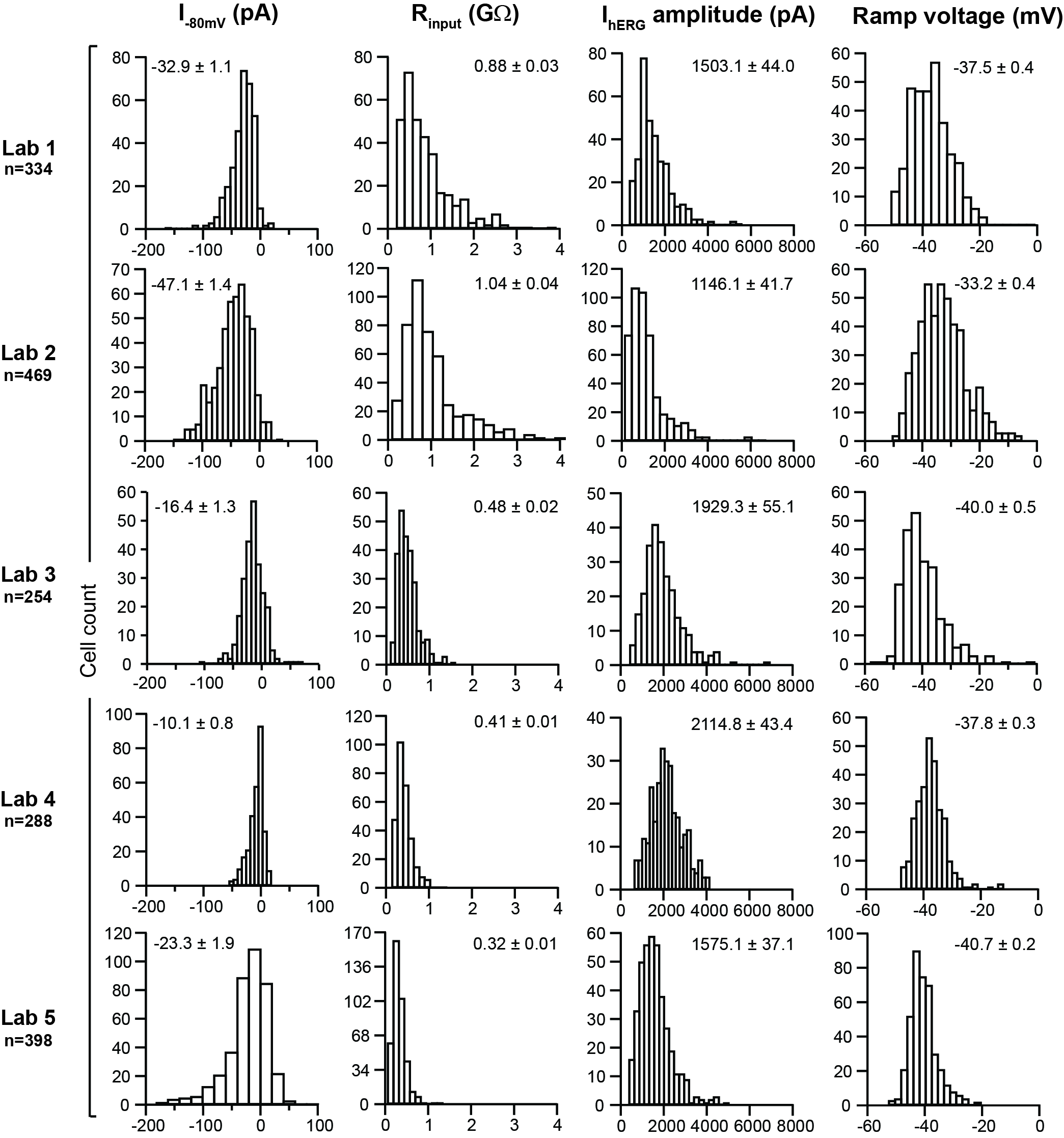

Supplement: Supplementary file 10 — Supplementary Material 10 [file 41598_2025_15761_MOESM10_ESM.tif]
